# Engineering selective amyloid precursor protein inhibitors by machine learning and deep mutational scanning

**DOI:** 10.1002/pro.70712

**Published:** 2026-07-20

**Authors:** Reut Meiri, Oz Reuveni, Michal Levi, Evette S. Radisky, Niv Papo, Yaron Orenstein

**Affiliations:** ^1^ Department of Computer Science and Artificial Intelligence Bar‐Ilan University Ramat Gan Israel; ^2^ Avram and Stella Goldstein‐Goren Department of Biotechnology Engineering Ben‐Gurion University of the Negev Beer‐Sheva Israel; ^3^ Department of Cancer Biology Mayo Clinic Comprehensive Cancer Center Jacksonville Florida USA; ^4^ National Institute of Biotechnology in the Negev Ben‐Gurion University of the Negev Beer‐Sheva Israel; ^5^ The Mina and Everard Goodman Faculty of Life Sciences Bar‐Ilan University Ramat Gan Israel

**Keywords:** deep mutational scanning, neural networks, next‐generation sequencing, protein engineering, protein–protein interactions

## Abstract

Deep mutational scanning (DMS) has proven effective for mapping protein–protein interactions (PPIs), but it cannot provide complete coverage of the mutation landscape, particularly for multi‐mutant variants. To address this limitation, we trained machine‐learning (ML) models on previously generated DMS data for a stabilized amyloid precursor protein inhibitor (APPI) binding to either of two serine proteases, mesotrypsin and kallikrein‐6 (KLK6), which are implicated in various human disorders. We combined the models to accurately predict the binding selectivity of APPI variants, including double‐mutant variants, for the two serine proteases. We achieved a Pearson correlation of 0.937 between predicted log_2_ selectivity enrichment ratios and DMS‐derived values. We further validated the predictions of our combined model by yeast‐surface‐display measurements and inhibition assays of purified APPI variants and revealed epistatic interactions that shape protease selectivity. Guided by binding selectivity predictions, we identified highly selective APPI variants, including the most selective mesotrypsin inhibitor reported to date. Together, these findings support the use of DMS and ML as a framework for predicting PPI selectivity and prioritizing selective therapeutic protein variants.

## 1 INTRODUCTION

Targeting the protein–protein interactions (PPIs) that facilitate and regulate various cellular processes poses significant challenges to the drug‐development community. Some PPIs are highly specific, while others lack specificity, involving proteins that engage multiple, often structurally similar, targets on overlapping interfaces (Nooren & Thornton, 2003). Whereas highly specific PPIs have been leveraged to develop protein‐based therapeutics with reduced off‐target toxicity, offering promising treatments for a variety of clinical conditions (Ebrahimi & Samanta, 2023; Slathia et al., 2023) engineering highly selective therapeutic proteins for closely related protein targets remains a far more difficult task, given their similar structural and chemical properties.

The binding specificity of proteins involved in non‐specific PPIs with a particular target may nonetheless be manipulated by mutating those proteins (Sharabi et al., 2014). Such mutations may alter the free energy of the PPI, thereby either destabilizing or strengthening the interaction (Jubb et al., 2017). PPI selectivity is shaped primarily by interface residues, where “hot‐spot” residues are interface positions that contribute significantly to binding free energy, such that mutations at hot‐spot positions typically reduce affinity, whereas “cold‐spot” residues are interface residues occupied by suboptimal amino acids, such that mutations at cold‐spot positions typically increase affinity (Fry & Vassilev, 2005; Shirian et al., 2016). In addition, correlated mutations can also affect binding specificity to particular targets (Pazos et al., 1997), as they represent co‐evolving residues that may be either in close proximity or separated from one another within a sequence. However, the combined effects of correlated mutations may not be simply additive, but rather epistatic (Starr & Thornton, 2016). The interactions between correlated mutations thus present challenges in protein engineering, as epistasis complicates predictions of binding affinity and selectivity outcomes (Miton & Tokuriki, 2016).

Two common high‐throughput techniques for assessing PPIs comprise yeast two‐hybrid screening (Coates & Hall, 2003) and yeast surface display (YSD) (Teymennet‐Ramírez et al., 2022). However, these techniques are time‐consuming, and their scalability is limited. Therefore, computational methods trained on high‐throughput datasets, such as those generated through deep mutational scanning (DMS), have gained significant attention. DMS generates rich datasets that capture the functional impact of mutations on proteins and can be used to probe PPIs (Fowler & Fields, 2014). Furthermore, integrating DMS with statistical approaches enables the identification of complex underlying patterns (Leander et al., 2022) and reveals the effects of mutations on PPIs. Nonetheless, despite the promise of high‐throughput approaches, DMS (which is based on data from experimental library screens) only partially covers the mutation space of a given protein. Since the search‐space size increases exponentially with the number of mutated residue positions, there is a combinatorial explosion of the number of possible protein variants (Currin et al., 2019). Thus, this issue of partial coverage constitutes a significant obstacle to progress in obtaining a comprehensive picture of the mutational landscape of PPIs (Livesey & Marsh, 2020).

To impute the mutational landscape of a specific PPI, a variety of computational methods have been developed to predict binding affinity based on the proteins' amino acid sequences. Among these methods, supervised methods have emerged as the most successful in predicting binding affinity (Livesey & Marsh, 2020). Supervised methods have advanced significantly with the development of deep neural networks, particularly convolutional neural networks (CNNs) (Gelman et al., 2021) and graph neural networks (Horne & Shukla, 2022). Still, many supervised methods are significantly less accurate at predicting beneficial mutations compared to deleterious mutations (Reeb et al., 2020). Their key limitation is their failure to capture epistatic effects, which are common across positions in a protein's amino acid sequence (Hsu et al., 2022). In addition, only very few studies have focused on selectivity prediction in the face of multiple targets, and therefore the challenge of engineering selective inhibitors remains to be addressed.

**FIGURE 1:**
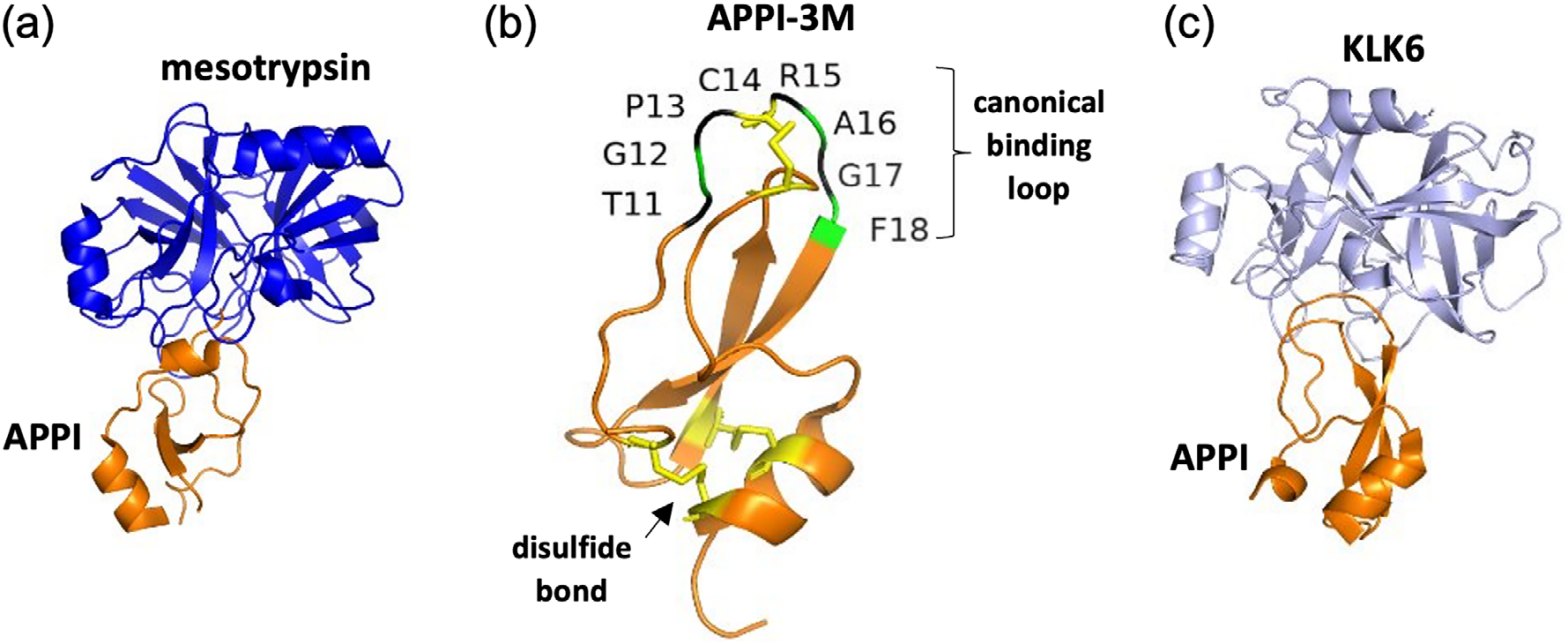
(a) Crystal structure of mesotrypsin complexed with APPI (PDB: 3L33) showing a molecule of mesotrypsin bound to a molecule of APPI. (b) Crystal structure of APPI‐3M (PDB: 5C67), which, like APPI, is stabilized by three disulfide bonds. (c) Crystal structure of KLK6 complexed with APPI (PDB: 5NX1) showing a molecule of KLK6 bound to a molecule of APPI.

To take up this challenge, we chose two homologous complexes as model systems; each comprises a complex of amyloid precursor protein inhibitor (APPI)—a human serine protease inhibitor belonging to the Kunitz family—with a serine protease, either mesotrypsin or kallikrein‐6 (KLK6), as the target protein (Scheidig et al., 1997) (Figure 1). The two proteases share high sequence homology and structural similarity, including the catalytic triad, His57, Asp102, and Ser195, that is highly conserved in serine proteases (Jiang et al., 2021). Both proteins constitute important targets for drug development: Mesotrypsin, normally expressed in the pancreas, brain, and colon (Salameh et al., 2010), is strongly implicated in tumor growth and the metastasis of epithelial cancers (Diederichs et al., 2004; Han et al., 2013; Hockla et al., 2010; Hockla et al., 2012; Jiang et al., 2010; Ma et al., 2015; Ma et al., 2019; Yang et al., 2008), and KLK6, normally expressed in the central nervous system (CNS), skin, and glands (Bayani & Diamandis, 2012), is associated with various cancers and neurodegenerative conditions (Bayani & Diamandis, 2012; Bayés et al., 2004; Blaber et al., 2007). Their similar structures constitute an obstacle to the development of high‐affinity selective inhibitors, as described below.

Wild‐type APPI (APPI_WT_) binds nonspecifically to the active site of serine proteases, including mesotrypsin and KLK6, via its canonical binding loop (spanning positions T11 to I18) (PDB:1ZJD). The positions in this loop are tolerant to amino acid substitutions (with the exception of C14, which maintains the integrity, stability, and structure of the loop), thereby enabling the production of APPI variants with a broad range of affinities toward the two targets (Cohen et al., 2016; Cohen et al., 2018; Naftaly et al., 2018; Sananes et al., 2018). Since APPI_WT_ is susceptible to cleavage by mesotrypsin (Cohen et al., 2016; Salameh et al., 2010), we worked, in this study, with a more stable variant of APPI, namely, APPI_M17G,I18F,F34V_, designated APPI‐3M, as the scaffold protein for engineering selective binding. This variant is more resistant to cleavage by mesotrypsin (Cohen et al., 2016; Sananes et al., 2018) and has a greater affinity (by two orders of magnitude) for mesotrypsin than for KLK6. It is therefore a particularly suitable scaffold for engineering specificity switches and designing selective binding for the two model protein targets.

As the first steps toward better understanding the mechanisms and evolution of selective serine protease‐inhibitor interactions and designing selective APPI inhibitors, we previously mapped the binding selectivity landscapes of APPI‐3M with mesotrypsin and KLK6 by using DMS and pairwise selectivity screening (Naftaly et al., 2018). However, that study focused on single mutants and estimated binding selectivity through affinity screens and retrospective analysis without computational modeling, thereby limiting our ability to rank double mutants or to predict the impact of single and combined mutations on selectivity. In our follow‐up study (Sarfati et al., 2022), we used machine‐learning (ML) with DMS data to predict APPI variant selectivity for mesotrypsin and KLK6, but the drawbacks of that study were that the predictions were most accurate at the extremes and less reliable for moderate selectivity, and validation with soluble proteins was lacking.

The current study was designed to address the above drawbacks: We trained ML models on our previously generated DMS data of APPI‐3M variants (Naftaly et al., 2018) with the aims to predict their binding selectivity for mesotrypsin and KLK6, to reveal epistatic interactions between mutated positions, and to highlight specific residues contributing to target selectivity. Thereafter, we used YSD to experimentally validate the predictions of the variants' selectivity. We then selected variants with pronounced selectivity toward either mesotrypsin or KLK6 for further purification and evaluation via target inhibition assays. Ultimately, we identified two highly selective inhibitors, one for mesotrypsin and the other for KLK6, that were not observed in the DMS data. The inhibitor of mesotrypsin is, to the best of our knowledge, the most selective mesotrypsin inhibitor vs. KLK6 reported to date.

## 2 METHODS

### 2.1 Computational analysis of the high‐throughput sequencing data

We previously generated the DMS data (Naftaly et al., 2018) used in this study, as follows. In brief, to select for binding specificity of APPI‐3M variants for mesotrypsin vs. KLK6, we subjected a YSD mutagenesis library of variants of APPI‐3M to sorting by FACS. We collected the presort library and two distinct fractions of the sorted population, namely, the fractions with preferential binding for mesotrypsin and KLK6. Three repetitions of each library fraction were analyzed by high‐throughput sequencing (HTS). In the current study, we downloaded the data from GEO accession number GSE289251 and processed it as follows. We located the start of each coding segment by searching for the first two codons of APPI‐3M (GAAGTT). We translated the coding segment of each sequence of the three above‐mentioned fractions into their respective amino acid sequences. We discarded sequences with zero copies in the presort library and retained sequences with the same length as APPI_WT_ that did not contain internal stop codons or non‐desired mutations (i.e., outside the binding interface). We excluded sequences with mutations outside the binding loop to minimize unintended epistatic effects that are not at the focus of this study.

We then counted the number of occurrences of each variant in each library and calculated the frequency of each variant *var*_*i*_ in the library *lib*, $f_{{\mathrm{var}}_{i},\mathrm{lib}}$, as:

(1) $$f_{{\mathrm{var}}_{i},\mathrm{lib}}=\frac{\#{\text{reads}}_{{\mathrm{var}}_{i},\mathrm{lib}}\;}{\sum_{j=1}^{N}\#{\text{reads}}_{{\mathrm{var}}_{j},\mathrm{lib}}}$$

where $\#{\text{reads}}_{{\mathrm{var}}_{i},\mathrm{lib}}$ is the number of reads of variant ${\mathrm{var}}_{i}$ in library lib out of N variants. Thereafter, based on the frequencies of each variant, we calculated the enrichment ratios (ERs) of each variant separately for the mesotrypsin and KLK6 libraries as follows:

(2) $${\mathrm{ER}}_{{\mathrm{var}}_{i},\text{mesotrypsin}}=\frac{f_{{\mathrm{var}}_{i},\text{mesotrypsin}}}{f_{{\mathrm{var}}_{i},\text{presort}}}$$

(3) $${\mathrm{ER}}_{{\mathrm{var}}_{i},\mathrm{KLK}6}=\frac{f_{{\mathrm{var}}_{i},\mathrm{KLK}6}}{f_{{\mathrm{var}}_{i},\text{presort}}}$$

where the ER is the ratio of the frequency of a given variant ${\mathrm{var}}_{i}$ in the mesotrypsin or KLK6 library to the frequency of the same ${\mathrm{var}}_{i}$ in the presort library.

We then calculated the selectivity ER as follows:

(4) $${\mathrm{ER}}_{{\mathrm{var}}_{i},\text{selectivity}}=\frac{{\mathrm{ER}}_{{\mathrm{var}}_{i},\text{mesotrypsin}}}{{\mathrm{ER}}_{{\mathrm{var}}_{i},\mathrm{KLK}6}}=\frac{f_{{\mathrm{var}}_{i},\text{mesotrypsin}}}{f_{{\mathrm{var}}_{i},\mathrm{KLK}6}}$$

which is the ratio of the frequency of a given variant ${\mathrm{var}}_{i}$ in the mesotrypsin library to the frequency of the same variant in the KLK6 library.

The measure of the strength of the interaction between mutations at residue positions i and j in a double mutant variant is termed the coupling energy, $∆∆G_{\mathrm{int}}$, which was previously described (Naftaly et al., 2018) as:

(5) $$∆∆G_{\mathrm{int}}=-\text{RTln}\left(\frac{{\mathrm{ER}}_{\mathrm{ij},\text{selectivity}}}{{\mathrm{ER}}_{i0,\text{selectivity}}\times {\mathrm{ER}}_{0j,\text{selectivity}}}\right)$$

where R is the gas constant, T is the absolute temperature, ${\mathrm{ER}}_{\mathrm{ij},\text{selectivity}}$ denotes the selectivity ER of the double mutant, ${\mathrm{ER}}_{i0,\text{selectivity}}$ and ${\mathrm{ER}}_{0j,\text{selectivity}}$ denote the corresponding single mutants, and 0 represents the APPI‐3M background at the respective position.

### 2.2 Machine‐learning training dataset

**FIGURE 2:**
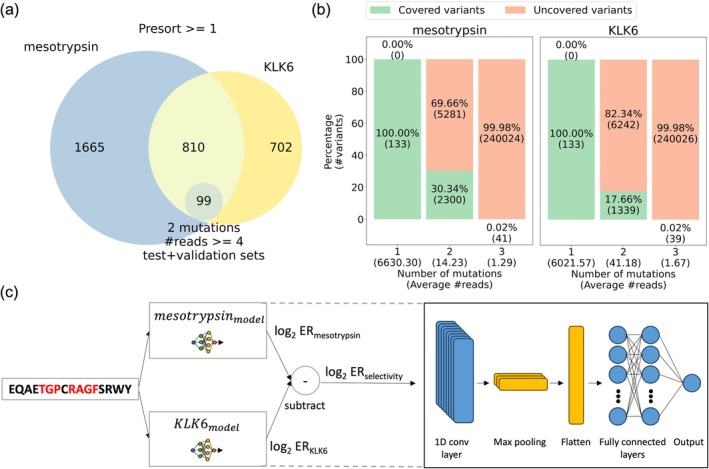
Data statistics and model architecture. (a) The overlap between mesotrypsin and KLK6 library fractions, where 99 variants in that overlap (≥4 repetitions) were used for test and validation. (b) Percentages of covered and uncovered variants with 1–3 mutations in the dataset. Exact counts and percentages are given, and average reads per variant are indicated below the plots. (c) Schematic representation of the CNN architecture used to predict the log_2_ ER values and the subtraction to predict the log_2_ selectivity ER.

By processing the raw DMS data, we generated a dataset containing the amino acid sequences, their three log_2_ ER labels (mesotrypsin, KLK6, and selectivity labels), and the log_2_ read count of each variant in each library. The dataset included 3177 variants. Of these, 2475 variants appeared in the mesotrypsin library and had a mesotrypsin log_2_ ER label, 1512 variants appeared in the KLK6 library and had a KLK6 log_2_ ER label, and 810 variants appeared in both libraries and had a selectivity label (Figure 2a). For each sequence, we retained the seven positions within the binding interface (positions 11, 12, 13, 15, 16, 17, and 18), as well as position 14 containing a cysteine residue which was not mutated. In addition, we included the four neighboring positions on each side of that region, which serve as padding for the CNN input, resulting in a total sequence length of 16. The dataset covered all 133 single‐mutation variants and included 3043 multiple‐mutation variants (Figure 2b).

### 2.3 Machine‐learning model architecture, input representation, and hyper‐parameters search

We explored multiple input representations and model architectures for predicting log_2_ ER values. Specifically, we compared five models: (1) a fully connected (FC) network with flattened one‐hot encoding, (2) an FC network using pretrained ESM2 embeddings extracted from the full‐length protein sequence, (3) an FC network combining one‐hot and ESM‐derived features, (4) a CNN with one‐hot encoding, and (5) a CNN with one‐hot encoding concatenated with ESM‐derived representations following the pooling layer. We did not compare more complex architectures since we were limited by the relatively small size of our dataset (3177 samples, where 2475 have a mesotrypsin log2 ER label, and 1512 have a KLK6 log2 ER label).

For ESM‐based representations, we evaluated residue‐level embeddings of 640 dimensions. This choice maintains a relatively small number of input features and model parameters, suitable for our dataset size. In addition, as reported before (Vieira et al., 2025), medium‐sized ESM‐2 models achieve strong performance, often comparable to larger models and in some DMS tasks even slightly outperforming them. We used the average difference in wild‐type and variant embeddings over the L positions:

(6) $$\Delta h=\frac{1}{L}\sum_{j=1}^{L}\left(h_{j}^{\mathrm{var}}-h_{j}^{3M}\right)$$

where $h_{j}^{\mathrm{var}}\in {\mathbb{R}}^{640}$ and $h_{j}^{3M}\in {\mathbb{R}}^{640}$ are the embedding vectors for position j in the variant and the APPI‐3M, respectively, produced by the ESM‐2 model.

We developed two models (one for each target) to predict log2 ER. We trained one model over the mesotrypsin labels, which we denoted ${\text{mesotrypsin}}_{\text{model}}$, and the other model over the KLK6 labels, which we denoted $\mathrm{KLK}6_{\text{model}}$ (Figure 2c). We denoted the subtraction of the output of the two models (the two predicted log_2_ ERs from ${\text{mesotrypsin}}_{\text{model}}$ and $\mathrm{KLK}6_{\text{model}}$) as the ${\text{selectivity}}_{\text{model}}$ prediction.

To train the models and to select the architecture and hyper‐parameters, we held‐out a high‐quality set of 99 variants that had two mutations, at least four reads in each of mesotrypsin and KLK6, and both mesotrypsin and KLK6 labels. We randomly partitioned this set into 50 variants as the validation set and 49 variants as the test set. For selecting the architecture and the hyper‐parameter values, we sampled 100 random hyper‐parameter combinations with a pre‐defined range for each parameter (Table S1). We selected the hyper‐parameter values that led to the highest Pearson correlation on the validation set of each model separately, and the architecture based on the ${\text{selectivity}}_{\text{model}}$ that achieved the best performance.

The final two models (one for each target) to predict log_2_ ER were ensembles of CNNs (Figure 2c). In each model, the input sequence is one‐hot encoded, resulting in a matrix of dimensions 20 × 16, where the order of amino acids is arbitrary and kept consistent across all training and evaluation data. This matrix serves as the input to a 1‐dimensional convolutional layer. The output from the convolutional layer passes through a max‐pooling layer with small pool sizes (2 for ${\text{mesotrypsin}}_{\text{model}}$ and 3 for $\mathrm{KLK}6_{\text{model}}$ as selected in the hyper‐parameter search), which reduces feature dimensionality and limits overfitting while preserving the strongest local activation signals. The resulting max‐pooled matrices are then flattened into a numerical vector. That vector is fed into two consecutive fully connected layers with a rectified linear unit (ReLU) activation function. The output from the second fully connected layer serves as the input to a single neuron with a linear activation function, producing the final network prediction.

We additionally implemented a multi‐task learning framework. In this model, convolutional layers are shared between tasks; this shared representation is fed to a two‐dimensional output layer, corresponding to the predicted two log2 ERs for mesotrypsin and KLK6 respectively. We trained the multi‐task model using the same training procedure and data splits as the single‐task models. To ensure a fair comparison, we performed a hyper‐parameter search over 100 values and selected the model achieving the best performance on the validation set.

We implemented the models using Keras Python library with TensorFlow backend (version 2.18.0).

### 2.4 Training and evaluation of the models

To increase model robustness, we used the random‐ensemble‐initialization approach, where we trained 100 models over the same data but with different randomly initialized weights (using standard Glorot initialization, the default for Conv1D and Dense layers in Keras/TensorFlow) and different random training data batches. In addition, so as to rely more on variants with a large statistical sample (a large number of reads), we used the log_2_ read counts of each variant in the mesotrypsin or KLK6 libraries as the sample weight in the training process. Then, for prediction, we obtained the final output by averaging the predictions of 100 trained models for each model type (i.e., ${\text{mesotrypsin}}_{\text{model}}$ or $\mathrm{KLK}6_{\text{model}}$). To derive the prediction for the ${\text{selectivity}}_{\text{model}}$, we subtracted the average prediction of the $\mathrm{KLK}6_{\text{model}}$ from that of the ${\text{mesotrypsin}}_{\text{model}}$. To assess confidence in the model's prediction, we calculated the coefficient of variation (CV) of a final prediction, which is an average of 100 predictions of the models in the ensemble, as follows:

(7) $$\begin{array}{rcl} \mathrm{CV} & = & \frac{\sqrt{\frac{1}{100}\sum_{i=1}^{100}{\left(M_{i}-\bar{M}\right)}^{2}+\frac{1}{100}\sum_{i=1}^{100}{\left(K_{i}-\bar{K}\right)}^{2}}}{\left|\frac{1}{100}\sum_{i=1}^{100}M_{i}-K_{i}\right|}\\ & = & \frac{\sqrt{{{\hat{\sigma }}_{\text{mesotrypsin}}}^{2}+{{\hat{\sigma }}_{\mathrm{KLK}6}}^{2}}}{\left|{\hat{\mu }}_{\text{selectivity}}\right|}=\frac{{\hat{\sigma }}_{\text{selectivity}}}{\left|{\hat{\mu }}_{\text{selectivity}}\right|} \end{array}$$

where *M* and *K* are the 100‐long prediction vectors of mesotrypsin and KLK6, respectively, $\hat{\sigma }$ is the estimated standard deviation, and $\hat{\mu }$ is the estimated mean. To evaluate the prediction performance of each model, we calculated the Pearson and Spearman correlation between the predictions and the experimental log_2_ ER values of a held‐out test set of 49 double‐mutant variants.

### 2.5 Leave‐one‐position‐out evaluation

To assess model robustness to predicting the effect of mutations at unobserved residue position and mitigate potential test–train leakage, we performed a leave‐one‐position‐out evaluation. In this evaluation, we held out all variants containing mutations at a given residue position (including both single and combinatorial mutations) as the test set. We then trained a model on all variants with mutations at the remaining six positions. We repeated this process for each position, ensuring that every residue position served as an independent test set. We evaluated model performance for mesotrypsin, KLK6, and selectivity predictions by Pearson and Spearman correlation coefficients.

### 2.6 Evaluation of prediction performance as a function of sequencing depth

To evaluate the ${\text{mesotrypsin}}_{\text{model}}$ and $\mathrm{KLK}6_{\text{model}}$ performance as a function of the sequencing depth, we used the same test set of 49 variants that had two mutations. We randomly selected 10 times a training set of X% (10 ≤ X ≤ 100) of the rest of the data (excluding 100% of the data with a fixed training set). After training of each model, we evaluated it in terms of the Pearson correlation between the predictions and the experimental log2 ER values of the test set. We repeated the training and evaluation process 10 times for each X, and report the mean and standard deviation of the Pearson correlation.

### 2.7 YSD assays for experimental validation

Given the resource‐intensive nature of producing soluble proteins and our need to test a large number of variants, we employed YSD as a preliminary selectivity screening technique to experimentally validate the ${\text{selectivity}}_{\text{model}}$ and to identify selective APPI variants (Figure S1). This technique allowed us to determine the binding levels of APPI variants to the two target proteases (in their soluble form) by displaying each variant on the surface of yeast cells in the presence of fluorescently labeled target proteases. The fluorescence signal thus served as a measure of the binding level of each displayed variant to the respective protease. We selected variants according to the following three categories, where different combinations of the single‐mutant variants formed the double‐mutant variants: (i) “single mutation‐observed,” which included variants with a single mutation (all of which were observed in the DMS data); (ii) “double mutations‐observed,” which included variants with two mutations that were observed in the DMS data; and (iii) “double mutations‐unobserved,” which included variants with two mutations that were not observed in the DMS data. The YSD technique and the construction and cloning of APPI variants in YSD format are described in the Data S1 (Methods).

To calculate the binding selectivity of each APPI protein variant, we divided the fluorescence intensity of that variant bound to a mesotrypsin‐FITC conjugate by the fluorescence intensity of the variant bound to KLK6‐APC conjugate on a logarithmic scale. To account for the variability in protein expression levels in the different APPI variants, we normalized the fluorescence intensities to the expression levels of each variant [determined using a phycoerythrin (PE)‐labeled anti c‐Myc antibody]. Additionally, to establish a reference protein for comparison of the selectivity of the different YSD variants, we used the APPI_P13Y,G17Y_ variant for further normalization, as it exhibited a moderate log_2_ selectivity ER and a moderate binding level for both targets and thus served as a good reference for comparison. We further normalized the fluorescence binding signals of all variants to the binding level of this variant. We then used the normalized data (Figure S2a–d) to calculate the selectivity index_YSD_ based on the YSD assay; this index reflects the preference of each APPI variant for binding to mesotrypsin vs. KLK6, as follows:

(8) $$\text{Selectivity}\;{\text{index}}_{\mathrm{YSD}}=\log_{2}\left(\frac{{\text{fluorescence}}_{\text{mesotrypsin}}/{\text{fluorescence}}_{\text{mesotrypsin, }\mathrm{ref}}}{{\text{fluorescence}}_{\mathrm{KLK}6}/{\text{fluorescence}}_{\mathrm{KLK}6,\mathrm{ref}}}\right)$$

where ${\text{fluorescence}}_{\text{mesotrypsin}}$ and ${\text{fluorescence}}_{\mathrm{KLK}6}$ are the fluorescence intensity of the variant obtained from binding to mesotrypsin‐FITC or KLK6‐APC conjugates, respectively, and ${\text{fluorescence}}_{\text{mesotrypsin},\mathrm{ref}}$ and ${\text{fluorescence}}_{\mathrm{KLK}6,\mathrm{ref}}$ are the fluorescence intensity of the reference variant APPI_P13Y,G17Y_ obtained from binding to the same conjugates.

### 2.8 Inhibition assays for experimental validation

We used the competitive inhibition equation (Equation 8) and the Morrison tight‐binding equation (Equation 9) to calculate the inhibition constant (*K*_i_) for each purified APPI variant:

(9) $$V=\frac{V_{\max}*\left[S\right]}{K_{m}*\left(1+\frac{\left[I\right]}{K_{i}}\right)+\left[S\right]}$$

where V is the formation rate of the cleavage product [p‐nitroaniline (pNA) for mesotrypsin and 7‐amino‐4‐methyl coumarin (AMC) for KLK6], v_max_ is the maximal rate of substrate (Z‐GPR‐pNA for mesotrypsin and BOC‐FSR‐AMC for KLK6) cleavage, *K*_m_ is the Michaelis constant, *K*_i_ is the inhibitor (APPI variant) constant, [S] is the substrate concentration, and [I] is the inhibitor concentration (Kakkar et al., 1999):

(10) $$\frac{V_{0}-V_{i}}{V_{i}}=\frac{{\left[I\right]}_{0}}{K_{i}*\left(1+\frac{{\left[S\right]}_{0}}{K_{m}}\right)}$$

where *V*_0_ is the steady‐state rate in the absence of the inhibitor, *V*_*i*_ is the steady‐state rate in the presence of the inhibitor, *K*_*m*_ is the Michaelis constant for substrate cleavage and [S]_0_ and [I]_0_ are the initial concentrations of the substrate and inhibitor, respectively. We performed calculations by using *K*_*m*_ values of 24.66 ± 1.30 μM for mesotrypsin and 329.30 ± 2.50 μM for KLK6 (Cohen et al., 2018).

We then calculated the selectivity index_inhibition_ of each purified variant as the logarithmic ratio of its *K*_i_ value against KLK6 to the *K*_i_ value for the same variant against mesotrypsin normalized to the corresponding *K*_i_ of APPI‐3M, as follows:

(11) $$\text{selectivity}\;{\text{index}}_{\text{inhibition}}=\log_{2}\left(\frac{\frac{K_{i},\mathrm{KLK}6}{K_{i_{3M}},\mathrm{KLK}6}}{\frac{K_{i},\text{mesotrypsin}}{K_{i_{3M}},\text{mesotrypsin}}}\right)$$

where *K*_i_,*KLK6* and *K*_i_,*mesotrypsin* are the inhibition constants of the variant for KLK6 and mesotrypsin, respectively, and *K*_*i3M*_,*KLK6* and *K*_*i3M*_,*mesotrypsin* are the inhibition constants of APPI‐3M for these proteases. Protein production and purification and the determination of the inhibition constant (*K*_i_) of APPI variants to mesotrypsin and KLK6 are described in Data S1 (Methods).

## 3 RESULTS

### 3.1 Training and evaluating ML models to predict the selectivity of APPI‐3M variants

We developed ML models to predict, for any given variant, the log_2_ selectivity ER, which is an experimental measurement correlated to the selectivity of the variant. We trained two models, designated ${\text{mesotrypsin}}_{\text{model}}$ and $\mathrm{KLK}6_{\text{model}}$, to predict the log_2_ ER values based on a specific library. The training dataset comprised single‐ and multi‐mutation variants (Figure 2b), along with their respective log_2_ ER labels (i.e., mesotrypsin and KLK6 labels). We held out 99 high‐quality variants, of which half were used for tuning hyper‐parameters and the other half were used for testing the trained model. We used the remaining dataset, consisting of single‐mutant and multiple‐mutant variants with lower read counts (Figure 2b) for training. During the training process, we employed the logarithmic value of the read count for each variant for sample weighting. We evaluated the performance of the ${\text{mesotrypsin}}_{\text{model}}$ and the $\mathrm{KLK}6_{\text{model}}$ in predicting log_2_ ERs in terms of the Pearson correlation of predicted vs. observed log_2_ ERs.

We evaluated five architectures differing in input representation and model type. For each architecture, we selected the best‐performing hyper‐parameter combination separately for ${\text{mesotrypsin}}_{\text{model}}$ and $\mathrm{KLK}6_{\text{model}}$ prediction, and the resulting models were used to compute ${\text{selectivity}}_{\text{model}}$ performance. The FC models with one‐hot, one‐hot combined with ESM2 representation, and ESM2‐only inputs achieved Pearson correlations of 0.920, 0.916, and 0.914 for the ${\text{selectivity}}_{\text{model}}$, respectively. The CNN models yielded Pearson correlations 0.929, and 0.927 for ${\text{selectivity}}_{\text{model}}$, for the one‐hot and one‐hot combined with ESM2 representation models, respectively (Figure S3). Overall, the CNN with one‐hot encoding achieved the highest performance across tasks. Accordingly, we selected the CNN with one‐hot input as our final model and used it in subsequent analyses. Moreover, most hyper‐parameter combinations for the CNN model led to high prediction performance (>0.88 Pearson corellation, Figure S4), demonstrating relative robustness to hyper‐parameter choices.

To assess whether shared representation learning across proteases could further improve performance, we additionally trained a multi‐task CNN in which shared convolutional layers feed a two‐dimensional output layer simultaneously predicting the two log2 ERs for mesotrypsin and KLK6. The multi‐task model achieved a Pearson correlation of 0.9287 for selectivity prediction, which was slightly lower than the corresponding single‐task CNN model (Pearson correlations 0.9291). Thus, while multi‐task learning is conceptually motivated by shared biochemical features of APPI interactions, it did not improve predictive performance in our dataset, and we chose not to use it in downstream analyses.

**FIGURE 3:**
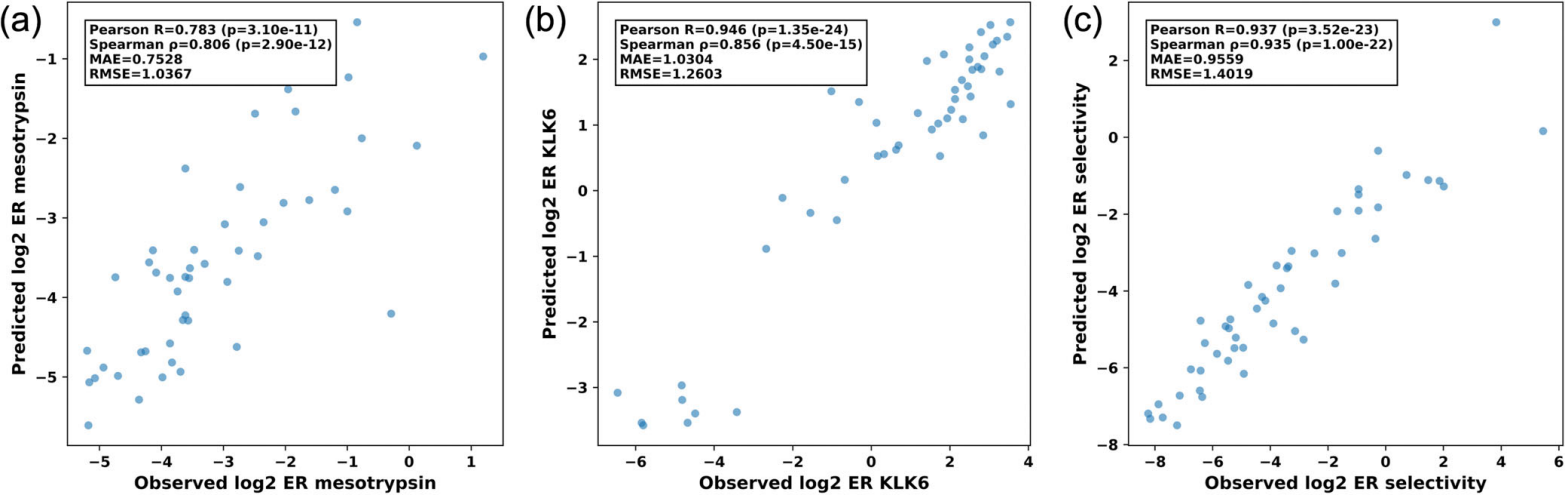
Predictive performance (correlation between predicted and observed log_2_ ERs) of the ML models for a held‐out high‐quality test set of 49 double‐mutant variants for (a) ${\text{mesotrypsin}}_{\text{model}}$, (b) $\mathrm{KLK}6_{\text{model}}$, and (c) ${\text{selectivity}}_{\text{model}}$.

Both the ${\text{mesotrypsin}}_{\text{model}}$ and the $\mathrm{KLK}6_{\text{model}}$ exhibited high performance on the held‐out test set (Figure 3a,b): Pearson correlations of 0.783 and 0.946, respectively (*p* values = 3.10e‐11 and 1.35e‐24, respectively) were obtained. In addition, strong rank‐based agreement was observed, with Spearman correlations of 0.806 and 0.856, respectively (*p* values = 2.90e‐12 and 4.50e‐15, respectively). Error‐based metrics further supported model performance, with mean absolute errors (MAE) of 0.753 and 1.030 and root mean square errors (RMSE) of 1.037 and 1.260 for the ${\text{mesotrypsin}}_{\text{model}}$ and the $\mathrm{KLK}6_{\text{model}}$, respectively. The ${\text{selectivity}}_{\text{model}}$, defined as ${\text{mesotrypsin}}_{\text{model}}$ ‐ $\mathrm{KLK}6_{\text{model}}$, also exhibited high performance on the held‐out test set (Figure 3c). A Pearson correlation of 0.937 (*P* value = 3.52e‐23) and a Spearman correlation of 0.935 (P value = 1.00e‐22) were observed between predicted and observed log ratio of selectivity ERs. The corresponding MAE and RMSE values were 0.956 and 1.402, respectively.

To further assess model robustness to predicting the effect of unobserved mutations and mitigating potential test–train leakage, we performed a leave‐one‐position‐out evaluation. Performance varied across positions, with high prediction performance observed for positions 11 and 13, where Pearson correlations for selectivity were 0.908 and 0.959, respectively. High performance was also observed for KLK6 predictions across most positions. However, performance was reduced for certain positions, such as 12, 15, and 17, where lower or negative correlations were observed for mesotrypsin and KLK6 predictions. Overall, while performance varied across positions, the model still demonstrated predictive capability under this challenging evaluation, suggesting some ability to generalize to unobserved mutations (Figure S5).

### 3.2 Effect of sequencing depth and sample weighing on the performance of the ML models

Next, we aimed to test how the sequencing depth influences the performance of our ML models. The sequencing depth affects the statistical sample size of each variant and consequently the robustness of the log2 ER values measured in the experiment. We assessed how the number of reads influences prediction performance of the models ${\text{mesotrypsin}}_{\text{model}}$, and $\mathrm{KLK}6_{\text{model}}$ by resampling in silico the whole dataset to generate simulated datasets with different numbers of DNA library sequencing reads. We trained the ${\text{mesotrypsin}}_{\text{model}}$, and $\mathrm{KLK}6_{\text{model}}$ on each simulated dataset and tested each model on the held‐out test set.

As expected, predictive performance generally improved with increasing dataset size for both proteases (Figure S6). This effect was most apparent for mesotrypsin, where the Pearson correlation increased from 0.504 using 10% of the data to 0.770 using the full dataset (slightly below the previously reported full dataset result due to a different random seed and number of shuffles). In contrast, KLK6 predictions remained consistently high even at lower sampling levels, with Pearson correlations above 0.887 across all sequencing depths. These results suggest that, despite incomplete sampling of the sequence space, the dataset contains sufficient information to support robust model training, while indicating that increased sequencing depth could further improve performance, particularly for mesotrypsin prediction.

To evaluate the impact of sample weighting based on sequencing depth, we compared three training schemes: (i) no sample weighting, (ii) raw read‐count‐based weighting, and (iii) log2‐transformed read‐count weighting (Figure S7). Overall, both the unweighted and log2‐weighted models achieved similarly high predictive performance, with average Pearson correlations of 0.9146 and 0.9159, respectively, on held‐out validation set. In contrast, using raw read counts as weights substantially degraded performance, particularly for mesotrypsin prediction (0.665 compared to 0.897 for the log2‐weighted model), indicating that untransformed counts overemphasize high‐coverage variants and impair generalization. These results suggest that while incorporating read depth information does not strongly improve predictive performance, log‐transformation is important for preventing extreme weighting effects.

### 3.3 Identifying positions that affect the selectivity of APPI for mesotrypsin vs. KLK6

To investigate the binding landscape of the interaction between APPI‐3M and each of the two target serine proteases, mesotrypsin and KLK6, we predicted, using the ${\text{selectivity}}_{\text{model}}$, all possible single‐mutant and double‐mutant log_2_ selectivity ERs. Given the predictions, we calculated the selective epistasis effects, that is, the deviation of a double‐mutant variant's predictions from the sum of its corresponding single‐mutant predictions. Positive selective epistasis is indicated when a combination of two mutations results in a change in the log_2_ selectivity ER ratio (mesotrypsin vs. KLK6) that is greater than the sum of the individual changes in selectivity, whereas negative selective epistasis is indicated when the combined effect is less than expected based on the sum of the individual effects.

**FIGURE 4:**
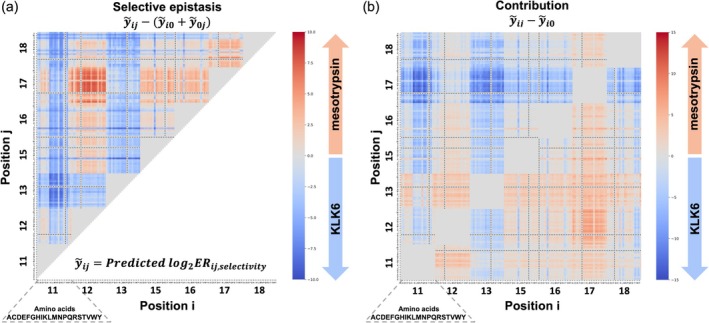
Analysis of double‐mutant vs. single‐mutant selectivity predictions. (a) Selective epistasis heatmap. (b) Double‐mutation selectivity heatmap. Each square is a 20 × 20 matrix containing the difference in predicted log_2_ selectivity ER values of double‐ and single‐mutant couplings between a mutation at position i and a mutation at position j.

We revealed that mutations at positions 12 and 17 produced the highest levels of positive selective epistasis (Figure 4a). Similarly, a mutation at position 12 combined with a mutation at position 15 or 16 yielded positive selective epistasis. In contrast, mutations at position 13 combined with mutations at another position resulted in negative selective epistasis, as did most mutations at position 11, where mutations at both positions 11 and 13 (cold spots for mesotrypsin) exhibited the highest levels of negative selective epistasis. A possible explanation for the pronounced negative epistasis, particularly in combinations involving two cold spots for mesotrypsin, is that individual mutations at these positions already yield high predicted selectivity for the target protease, and therefore combining them does not enhance selectivity beyond the sum of their individual effects.

Additionally, we calculated the contribution of each position and amino acid residue to the selectivity of double‐mutant variants for one of the two protease targets (Figure 4b). The results confirmed previous findings and demonstrated that adding a second mutation at position 13 consistently increased selectivity for mesotrypsin. Likewise, most substitutions at position 11 led to enhanced selectivity for mesotrypsin. Conversely, mutations at position 17 consistently increased selectivity for KLK6. These findings align with the known roles of position 13 as a cold spot for mesotrypsin and position 17 as a cold spot for KLK6 (Naftaly et al., 2018). Furthermore, these selectivity‐switch residues (13 and 17) enable a shift in selectivity from one protease to the other (mesotrypsin to KLK6 or vice versa) (Naftaly et al., 2018).

### 3.4 Selecting candidate variants for experimental validation

The next step was to experimentally validate, by using YSD, our ${\text{selectivity}}_{\text{model}}$ in predicting double‐mutant variants, followed by analysis of the predicted selective epistasis effects and residue contributions to selectivity. For this experimental validation, we selected 42 variants, including 20 double‐mutant variants, 21 single‐mutant variants corresponding to the double‐mutant variants, and the APPI‐3M scaffold variant (Table S2). The double‐mutant variants were selected on the basis of two criteria: (i) known cold‐spot selectivity‐switch positions (11 and 13 for mesotrypsin, and 17 and 18 for KLK6) (Naftaly et al., 2018) and their combinations in two mutations, and (ii) variants with double mutations that exhibited relatively high selective epistasis. Seventeen out of the 20 double‐mutant variants awarded high‐confidence predictions (i.e., a CV smaller than 1.5), except for three variants where the absolute predicted value was smaller than 1, leading to a higher CV (Figure S8).

### 3.5 Experimental validation by a YSD screen

**FIGURE 5:**
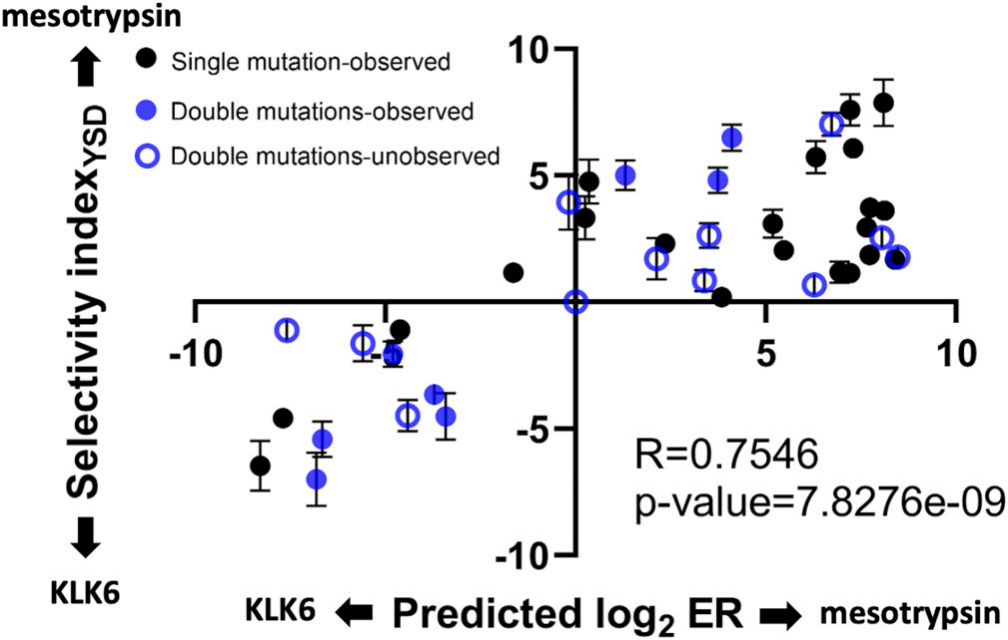
Experimental validation of selectivity predictions based on YSD measurements. Correlation of log_2_ selectivity ER, as estimated by the ${\text{selectivity}}_{\text{model}}$, with the selectivity index_YSD_ determined using the YSD assay.

We used YSD as a preliminary selectivity screening technique to test the utility of the ${\text{selectivity}}_{\text{model}}$ for the identification selective single or double APPI variants, both for variants observed in the DMS data and for unobserved variants. The screening step revealed that predicted log_2_ selectivity ER values obtained from the ${\text{selectivity}}_{\text{model}}$ correlated well with the selectivity index_YSD_ (Figure 5) (Pearson correlation 0.755; *P* value =7.828e‐09). Specifically, most variants predicted to exhibit selectivity toward KLK6 or mesotrypsin did indeed consistently demonstrate such selectivity. Notably, the ${\text{selectivity}}_{\text{model}}$ accurately predicted the selectivity preferences of double‐mutant variants that were not observed in the DMS data, emphasizing the model's generalizability capabilities. However, two variants with intermediate log_2_ selectivity ER values that were predicted to exhibit selectivity to KLK6 showed slightly stronger binding to mesotrypsin, thereby suggesting that the $\text{selectivit}y_{\text{model}}$ is less accurate for some variants predicted with moderate selectivity scores. In addition, the ${\text{selectivity}}_{\text{model}}$ showed some inconsistency in predicting the exact level of selectivity, with a number of variants that had been predicted to be highly selective for either mesotrypsin or KLK6 showing instead only a moderate level of selectivity by YSD.

To evaluate how well the model predicts selective epistasis effects, we compared experimentally measured selective epistasis values (based on YSD measurements) with selective epistasis inferred from ${\text{selectivity}}_{\text{model}}$ predictions (Figure S9). Overall, the ${\text{selectivity}}_{\text{model}}$ captured the qualitative selective epistatic landscape, correctly reproducing the selective epistatic trend in 8 out of 10 double‐mutant variants. Notably, APPI_G17Y,F18K_ and APPI_G17Y,F18R_ both involving the same substitution at position 17, were predicted in the wrong direction. However, ${\text{selectivity}}_{\text{model}}$ overestimated the magnitude of epistatic effects in several cases, as reflected by longer deviations between predicted and measured values. For example, variant such as APPI _T11K,P13I_ and APPI_T11M,P13D_ exhibit strong negative epistasis experimentally, which was correctly identified in direction but somewhat exaggerated in magnitude by the model. These results indicate that while the ${\text{selectivity}}_{\text{model}}$ epistasis is not perfectly calibrated quantitatively, it provides a reliable qualitative approximation of experimentally observed mutational interactions.

### 3.6 Experimental validation by inhibition assays

We followed our preliminary YSD screen by validation of the binding selectivity using purified soluble APPI variants. To confirm the selectivity of single‐ and double‐mutant variants through inhibition assays, we chose highly selective APPI variants based on two criteria: (i) highest or lowest predicted log_2_ selectivity ER values, and (ii) highest or lowest selectivity index_YSD_ scores (Figure S2a,b). In addition, we also produced and purified single‐ and double‐mutant variants that exhibited both intermediate log_2_ selectivity ER values and intermediate selectivity index_YSD_ scores (Figure S2c,d). The APPI‐3M scaffold and 10 of the 18 selected variants had been produced in previous work, and their *K*_i_ inhibition constants were therefore available (Cohen et al., 2016; Naftaly et al., 2018). The production and purification procedure for APPI variants and *K*_i_ measurements are described under Supplementary Results and shown in Figures S10–S13.

**FIGURE 6:**
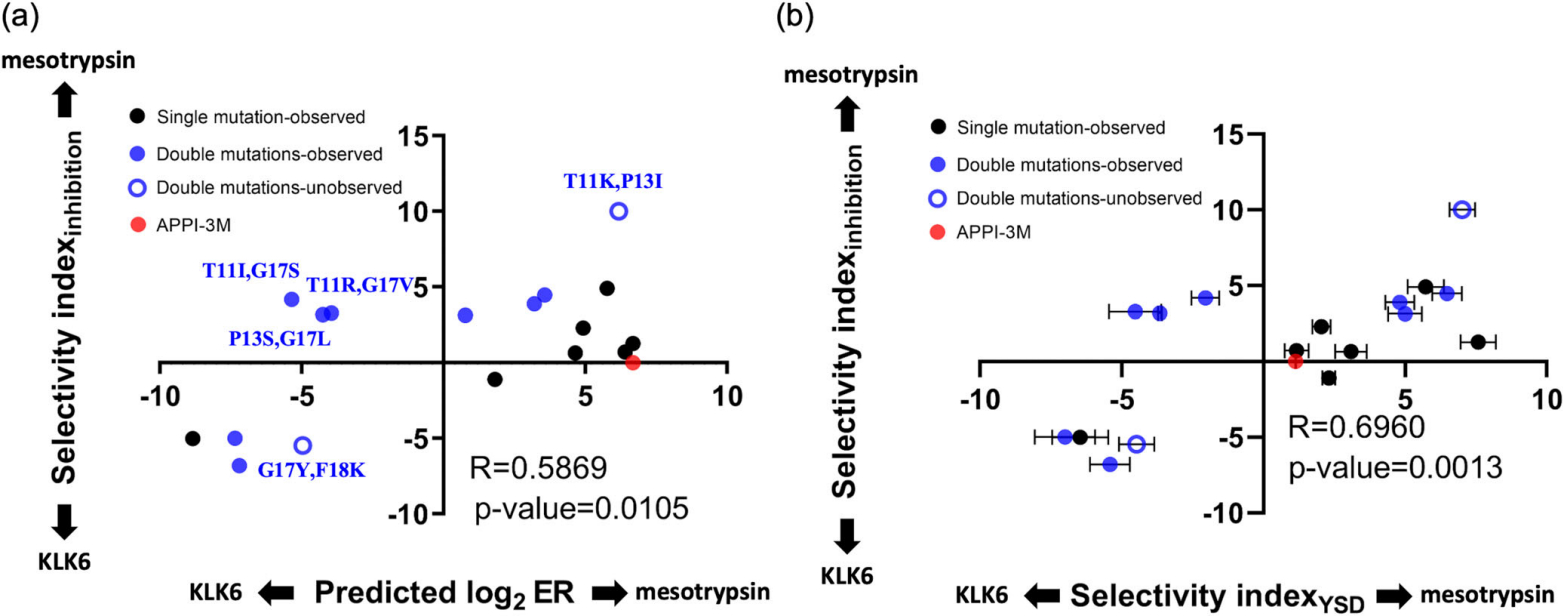
Validation of selectivity prediction determined via the inhibition constants obtained for purified vs. YSD APPI variants. (a) Correlation between the selectivity index_inhibition_ determined by affinity (*K*_i_) constants of the purified proteins and selectivity log_2_ ER determined from DMS, followed by prediction by the ${\text{selectivity}}_{\text{model}}$. (b) Correlation between the selectivity index_YSD_, determined by YSD and the selectivity index_inhibition_ based on the *K*_i_ values of purified variants.

The log_2_ selectivity ER predictions correlated well with the selectivity index_inhibition_ derived from the *K*_i_ values of the purified proteins, including both observed and unobserved variants (Figure 6a,b). However, the ${\text{selectivity}}_{\text{model}}$ was less accurate for some variants predicted with intermediate log_2_ selectivity ER values, such as APPI_T11I,G17S_, APPI_P13L,G17S_ and APPI_T11R,G17V_, compared to extreme selectivity ER values. Those three double‐mutant variants were predicted to exhibit moderate selectivity, with a preference for KLK6 but, in practice, showed a stronger affinity for mesotrypsin when purified (Figure 6a). Similarly to the results obtained from selectivity index_YSD_ derived from YSD measurements, these results may suggest that the predictions of the ${\text{selectivity}}_{\text{model}}$ for variants with moderate log_2_ selectivity ER values are less accurate than those for variants with a strong preference for one target vs. the other. This reduced accuracy may result from the design of the sorting experiment, in which only variants showing clear binding preference for either mesotrypsin or KLK6 were collected. As a result, variants with no preference or only moderate selectivity were underrepresented in the training dataset.

The selectivity index_YSD_ derived from YSD measurements of APPI variants precisely reflected the selectivity predictions, yielding a Pearson correlation of 0.8728 (*P* value = 2.2971e‐06) (Figure S14b). In contrast, the *K*_i_‐based measurements of purified APPI variants yielded a lower Pearson correlation of 0.5869 (*P* value = 0.0105) (Figure 6a). The superior precision of the YSD‐based determination of selectivity is likely attributable to the experimental setup for the YSD‐based measurements and the DMS (used as an input to the ${\text{selectivity}}_{\text{model}}$ to obtain log_2_ selectivity ER values): in the YSD set‐up, the two target proteases—mesotrypsin and KLK6—are both present in the reaction tube [thereby mimicking the competitive binding observed during the DMS (Naftaly et al., 2018)] and interact with the APPI variant immobilized to a surface. This experimental setup differs from that for the *K*_i_ measurements in which the soluble form of the APPI variant competes with a single protease substrate (either mesotrypsin or KLK6) for binding at its active site.

Variants with predominantly selective binding to mesotrypsin included those with mutations at position 13, while mutations at position 17 enhanced selectivity for KLK6 (Figure 6, Table S3). Examples include APPI_T11K,P13I_ and APPI_G17Y,F18K_ with binding preferences for mesotrypsin and KLK6, respectively (Figure 6a). These variants were not observed in the DMS data (i.e., were not used for ${\text{selectivity}}_{\text{model}}$ training) and were thus predicted solely by the ${\text{selectivity}}_{\text{model}}$. APPI_T11K,P13I_ exhibited tight‐binding inhibition behavior with mesotrypsin, with a *K*_i_ value of 1.8 ± 1.0 nM (Figure S12h), and exemplified a classic inhibition model against KLK6, with a *K*_i_ constant of 6.9 ± 1.2 μM (Figure S13c). This *K*_*i*_ value translates into a 3832‐fold increase in selectivity for mesotrypsin over KLK6 (Table S3) and also indicates a 2254‐fold increase in selectivity compared to APPI_WT_ (Cohen et al., 2018). To the best of our knowledge, APPI_T11K,P13I_ is the most selective mesotrypsin inhibitor vs. KLK6 reported to date. The APPI_G17Y,F18K_ variant also exhibited classic inhibition behavior with mesotrypsin, with a *K*_i_ value of 458.6 ± 46.0 nM (Figure S12g) and a classic inhibition model with KLK6 with a *K*_i_ constant of 38.0 ± 7.9 nM (Figure S13f). This *K*_*i*_ value represents a 12.1‐fold increase in selectivity for KLK6 over mesotrypsin (Table S3). The above findings, obtained for variants carrying two mutations that were not observed in the DMS data, are in agreement with the ${\text{selectivity}}_{\text{model}}$ predictions.

## 4 DISCUSSION

In this study, we developed an ML‐based method to predict the selectivity of multi‐mutation APPI variants for homologous serine protease targets across a broad range of binding affinities: By leveraging deep neural networks trained on our previously generated DMS data for the scaffold protein APPI‐3M, we predicted the selectivity ER of APPI variants for mesotrypsin vs. KLK6. We followed the computational prediction by experimental validation using APPI protein variants produced on a YSD platform. This platform provided a rapid and resource‐efficient solution for identifying highly selective protein‐based inhibitors, allowing us to prioritize promising protein variants for further purification and evaluation via target inhibition assays. The combined computational‐experimental methodology accurately predicted binding selectivity and selective epistatic interactions, enabling the identification of novel APPI variants that were not observed in the original library. Among those variants were highly selective inhibitors for mesotrypsin and KLK6, including the most selective mesotrypsin inhibitor vs. KLK6 reported to date.

One of the key findings of our study was the identification of specific amino acid positions, particularly positions 11, 13, 17, and 18, that play a critical role in determining target selectivity. Mutations at positions 11 and 13, which are cold spots for mesotrypsin, enhanced selectivity for mesotrypsin, while mutations at positions 17 and 18 increased selectivity for KLK6. This position‐specific influence on selectivity was supported by both our ${\text{selectivity}}_{\text{model}}$ and experimental validation via YSD and inhibition assays.

Another major novelty of this work was the demonstration of non‐additive, epistatic effects of mutations on selectivity. By comparing predicted selectivity values of double‐mutant variants to the summed effects of their single mutations, we uncovered multiple cases of both positive and negative selective epistasis. For example, combinations involving positions 12 and 17 showed strong positive epistasis, while combinations involving positions 11 and 13, each already known as cold spots for mesotrypsin, exhibited negative epistasis. These non‐additive interactions highlight the necessity of taking into consideration both the presence of multiple mutations and epistasis in selectivity prediction and protein engineering.

Furthermore, our results showed that epistatic interactions are not rare exceptions in the mutational landscape and that they play a crucial role in determining the functional outcome of mutations. Models that do not take these effects into consideration may miss promising mutation combinations or wrongly predict how engineered variants will behave. In our case, the ability to detect and quantify selective epistasis allowed us to rationally select and validate double‐mutant variants with enhanced or shifted target specificity.

The promising results described above notwithstanding, the study has several limitations. First, while our ${\text{selectivity}}_{\text{model}}$ performed well in predicting selectivity, its performance was less successful for variants with moderate selectivity scores compared to scores at the high or low extremes. This outcome suggests potential biases in the training dataset, with fewer variants representing intermediate selectivity regimes, as seen in the comparison between DMS measurements and *K*_i_‐based inhibition constants (Figure S14a). From a protein design perspective, this limitation implies that the model is most reliable for identifying variants with strongly enhanced or diminished selectivity, which are often of primary interest in early‐stage screening. In addition, while multiple targets may exist in a cellular environment, the current study focuses on selectivity prediction between only two proteases. Still, it is a step toward ML‐based prediction of protein selectivity in more complex cellular environments as most studies focus only on a single protease. Second, we validated our predictions primarily by using YSD, which, while effective, may not fully reproduce the conditions of soluble protein interactions. The differences between YSD‐based selectivity indexes and *K*_i_‐based inhibition constants (Figure 6b) highlight the need for further improvement in the experimental validation techniques. Third, our study focused on double‐mutant variants, leaving the combinatorial effects of higher‐order mutations unexplored. Future studies focusing on variants with more than two mutations will be necessary to fully capture the complexity of these APPI‐serine protease PPIs.

In light of the above, we aim to expand our study in several directions. To improve predictive performance for *K*_i_ inhibition constants, we plan to develop models based on more advanced ML techniques—advanced architectures, such as graph neural networks, and unsupervised learning techniques, including large protein language models (Livesey & Marsh, 2023). Additionally, we will expand the training dataset to include a broader range of multiple‐mutant variants to enhance model generalizability. Integrating data from YSD validation as part of a transfer‐learning strategy could further improve predictions, as our analysis indicates that YSD‐derived selectivity indexes are better correlated with *K*_*i*_ inhibition constants than current model predictions. Furthermore, cellular experimental validation followed by in‐vivo assays will be crucial in assessing the therapeutic potential of the designed inhibitors. More generally, we plan to extend this approach to additional protein families and inhibitor scaffolds to assess how broadly it can support the prediction of PPI selectivity.

Given the inherent challenges of distinguishing selectivity for two protease targets with similar structural characteristics and predicting the effects of multiple mutations on protein selectivity, our findings underscore the combined effectiveness of YSD screening and ML‐driven predictions in discovering potent double‐mutant inhibitors that specifically target highly conserved regions of serine proteases. More generally, by reducing the need for extensive experimental screening, our ML‐based approach can accelerate the discovery of novel protein variants with optimized selectivity and binding affinity. This methodology could be extended beyond protease inhibitors to other protein classes, including antibodies, unlocking significant therapeutic potential.

## AUTHOR CONTRIBUTIONS

**Reut Meiri:** Data curation; investigation; formal analysis; visualization; writing – review and editing; writing – original draft. **Niv Papo:** Conceptualization; methodology; formal analysis; supervision; funding acquisition; visualization; project administration; writing – review and editing; writing – original draft. **Evette S. Radisky:** Conceptualization; funding acquisition. **Yaron Orenstein:** Conceptualization; methodology; formal analysis; supervision; project administration; visualization; funding acquisition; writing – original draft; writing – review and editing. **Oz Reuveni:** Data curation; investigation; validation; formal analysis; writing – original draft; writing – review and editing; visualization. **Michal Levi:** Data curation; investigation; validation; formal analysis; visualization; writing – review and editing; writing – original draft.

## CONFLICT OF INTEREST STATEMENT

The authors declare no competing interests.

## Supporting information


**FIGURE S1.** Selectivity determination using yeast surface display (YSD). (a) Schematic drawing of APPI variant binding measurements using the YSD system. The APPI variant was displayed on the yeast cell surface in the presence of labeled proteases, with mesotrypsin labeled with FITC and KLK6 labeled with APC. (b) Detection of variant binding to the two protease targets. (c) Determination of variant expression using a fluorescent anti‐c‐Myc antibody labeled with PE.
**FIGURE S2.** Binding determination using a YSD preliminary screen. Fluorescence intensity of variants in response to binding with fluorescently labeled proteases is shown. To prevent bias due to variable protein expression levels on the yeast surface, intensity values were normalized to the expression level of each variant (to obtain intrinsic binding values) and to the intrinsic binding of the reference variant APPI_P13Y,G17Y_, which exhibited moderate binding levels for both targets. Variants were grouped into four categories according to high or intermediate selectivity to mesotrypsin or KLK6, as determined by their predicted log_2_ selectivity ER values: (a) selective single mutants, (b) selective double mutants, (c) intermediate single mutants, and (d) intermediate double mutants. Black dashed lines indicate variants whose selectivity as soluble proteins was assessed via inhibition assays.
**FIGURE S3.** Comparison of machine‐learning models for selectivity prediction. Pearson correlation coefficients of the ${\text{selectivity}}_{\text{model}}$ on the validation set for five architectures: fully connected (FC) and convolutional‐neural‐network (CNN) models using different input representations (one‐hot encoding, ESM2 embeddings, and their combination). We trained each model using hyper‐parameters selected separately by random search for mesotrypsin and KLK6 prediction. We report results on the held‐out validation set of 50 variants containing two mutations.
**FIGURE S4.** Distribution of CNN model performance across hyper‐parameter combinations. Histogram of Pearson correlation values for the ${\text{selectivity}}_{\text{model}}$ obtained from 100 randomly sampled hyper‐parameter combinations.
**FIGURE S5.** Leave‐one‐position‐out evaluation of model performance. Bar plots show Pearson and Spearman correlation coefficients for ${\text{mesotrypsin}}_{\text{model}}$, $\mathrm{KLK}6_{\text{model}}$, and ${\text{selectivity}}_{\text{model}}$ predictions when all variants containing mutations at a given position were excluded from training and used only for testing.
**FIGURE S6.** Prediction performance as a function of the size of the sequencing depth. We report results on the held‐out test set of 49 variants containing two mutations. A training set of 10%–100% of the rest of the data was randomly selected 10 times (excluding 100% of the data with a fixed training set). The library size of the training sets is shown as the mean ± standard deviation of 10 repeats.
**FIGURE S7.** Effect of sample weighting strategy on model performance. Predictive performance (correlation between predicted and observed log2 ERs) of three sample weighting conditions: log2 read count, raw read count, and no weights. We report results on the held‐out validation set of 50 variants containing two mutations.
**FIGURE S8.** Heat map matrix of coefficients of variation for double mutations. Each square is a 20 × 20 matrix containing coefficient of variation values for each prediction of coupling between a mutation at position i with a mutation at position j.
**FIGURE S9.** Comparison of measured and predicted epistasis in double mutants. For each double mutant variant, experimentally measured selective epistasis (purple) is compared to ${\text{selectivity}}_{\text{model}}$ predicted selective epistasis (green), with connecting lines indicating the deviation between them. The dashed line at zero separates positive and negative selective epistasis.
**FIGURE S10.** Purification process for the APPI variants. (a–e) Examples of the purification procedure for APPI_T11R,G17V_, where (a) nickel affinity chromatography purification profile; (b) SDS‐PAGE analysis of fractions post affinity chromatography; (c) size‐exclusion chromatography (SEC) profile, showing absorbance (black) and conductivity (blue) curves; and (d, e) SDS‐PAGE analysis of fractions post SEC. (f) SDS PAGE of all the purified APPI variants. Variant APPI_T11K,P13I_ was designed such that it did not include a FLAG tag.
**FIGURE S11.** Mass spectrometry analysis of APPI variants. A.U. denotes Arbitrary Units, representing relative light intensity. The displayed peaks represent the average molecular weights (Mw) of each variant, given in daltons. The theoretical and experimental molecular weights of each APPI variant showed high similarity, confirming the correct molecular mass of the purified variant.
**FIGURE S12.** Kinetics of mesotrypsin inhibition by APPI. (a–g). Mesotrypsin cleavage of the peptide substrate Z‐GPR‐pNA is competitively inhibited by APPI variants. (H) The *K*_i_ for APPI_T11K,P13I_ was calculated using the tight‐binding model (Morrison equation). The velocity of product formation at the beginning of the reaction was determined from the increase in absorbance (410 nm) caused by the release of pNA upon mesotrypsin cleavage.
**FIGURE S13.** Kinetics of KLK6 inhibition by APPI. (a–f) KLK6 cleavage of peptide substrate Boc‐FSR‐AMC is competitively inhibited by APPI variants. (g, h) The *K*_*i*_ values for APPI_T11R,G17V_ and APPI_P13L,F18L_, respectively, were calculated using the tight‐binding model (Morrison equation). The velocity of product formation at the start of the reaction was determined from the increase in fluorescent signal caused by the release of AMC upon KLK6 cleavage.
**FIGURE S14.** Performance of selectivity prediction. (a) Correlation between *K*_*i*_ constants ratio for selected variants and their selectivity log_2_ ER values as determined from DMS data. (b) Correlation of log_2_ selectivity ER, as predicted by the ${\text{selectivity}}_{\text{model}}$, with selectivity index_YSD_ being determined from fluorescence intensity binding signals using the YSD platform and FACS.
**TABLE S1.** Search range for hyper‐parameter values.
**TABLE S2.** Selected APPI variants.
**TABLE S3.** Summary of selected variants kinetics, predicted log_2_ selectivity ER, and selectivity index (variants in bold are discussed in the main text).
**TABLE S4.** Oligonucleotides used for site‐directed mutagenesis of APPI variants in the pCTCON plasmid.
**TABLE S5.** Oligonucleotides used in site‐directed mutagenesis for double mutation APPI variants.
**TABLE S6.** Oligonucleotides used to add NheI and BamHI restriction sites to the edges of the APPI gene sequence.
**TABLE S7.** Oligonucleotides used to add the APPI gene sequence edges with EcoRI and AvrII restriction sites.

## Data Availability

Unprocessed FASTQ files were obtained from the GEO under accession number GSE289251. Code and processed data used in this study are available at https://github.com/OrensteinLab/APPI-KLK6-Meso and have been archived at 10.5281/zenodo.15823190.
