# Supplementary material for: Engineering selective amyloid precursor protein inhibitors by machine learning and deep mutational scanning
Source: Protein Sci. 2026 Jul 20;35(8):e70712. doi: 10.1002/pro.70712 (PMC13385218; doi:10.1002/pro.70712)
Supplement: Supplementary file 1 — FIGURE S1. Selectivity determination using yeast surface display (YSD). (a) Schematic drawing of APPI variant binding measurements using the YSD system. The APPI variant was displayed on the yeast cell surface in the presence of labeled proteases, with mesotrypsin labeled with FITC and KLK6 labeled with APC. (b) Detection of variant binding to the two protease targets. (c) Determination of variant expression using a fluorescent anti‐c‐Myc antibody labeled with PE. FIGURE S2. Binding determination using a YSD preliminary screen. Fluorescence intensity of variants in response to binding with fluorescently labeled proteases is shown. To prevent bias due to variable protein expression levels on the yeast surface, intensity values were normalized to the expression level of each variant (to obtain intrinsic binding values) and to the intrinsic binding of the reference variant APPIP13Y,G17Y, which exhibited moderate binding levels for both targets. Variants were grouped into four categories according to high or intermediate selectivity to mesotrypsin or KLK6, as determined by their predicted log2 selectivity ER values: (a) selective single mutants, (b) selective double mutants, (c) intermediate single mutants, and (d) intermediate double mutants. Black dashed lines indicate variants whose selectivity as soluble proteins was assessed via inhibition assays. FIGURE S3. Comparison of machine‐learning models for selectivity prediction. Pearson correlation coefficients of the selectivitymodel on the validation set for five architectures: fully connected (FC) and convolutional‐neural‐network (CNN) models using different input representations (one‐hot encoding, ESM2 embeddings, and their combination). We trained each model using hyper‐parameters selected separately by random search for mesotrypsin and KLK6 prediction. We report results on the held‐out validation set of 50 variants containing two mutations. FIGURE S4. Distribution of CNN model performance across hyper‐parameter com [file PRO-35-e70712-s001.docx]

**Supplementary Information**

**Engineering Selective Amyloid Precursor Protein** **Inhibitors by Machine Learning and Deep Mutational Scanning**

Reut Meiri^1,†^, Oz Reuveni^2,†^, Michal Levi^2,†^, Evette S. Radisky^3^, Niv Papo^2,4,*^ and Yaron Orenstein^1,5,*^

^1^ Department of Computer Science, Bar-Ilan University, Ramat Gan, Israel

^2^ Avram and Stella Goldstein-Goren Department of Biotechnology Engineering, Ben-Gurion University of the Negev, Beer-Sheva, Israel

^3^ Department of Cancer Biology, Mayo Clinic Comprehensive Cancer Center, Jacksonville, Florida, USA

^4^ National Institute of Biotechnology in the Negev, Ben-Gurion University of the Negev, Beer-Sheva, Israel.

^5^ The Mina and Everard Goodman Faculty of Life Sciences, Bar-Ilan University, Ramat Gan, Israel

*To whom correspondence should be addressed. Email: [yaron.orenstein@biu.ac.il](mailto:yaron.orenstein@biu.ac.il); [papo@bgu.ac.il](mailto:papo@bgu.ac.il)

^†^The first three authors contributed equally to this work.

**Supplementary Methods**

**Construction and cloning of APPI variants in YSD format**

APPI variants were constructed using one of two methods. The first method involved site-directed mutagenesis of the human cDNA encoding the APPI-3M variant ^1^ in the pCTCON plasmid. The plasmid includes four restriction sites and two epitope tags, namely, a hemagglutinin antigen (HA) tag and c-Myc tag, arranged in the following sequence: EcoRI-Aga2-Xa-HA-(G_4_S)_3_ linker-NheI-APPI-BamHI-LPDKPLAFQDPS linker-c-Myc-XhoI. The pCTCON plasmid template containing the APPI-3M variant was amplified by PCR using Phusion DNA polymerase (New England Biolabs, Ipswich, MA, USA) and primers for mutagenesis (Integrated DNA Technologies, San Jose, CA, USA). The primers are listed in **Supplementary Table S4**. The second method employed restriction-ligation cloning in which APPI variants, originally included in pPIC9K (Invitrogen, Waltham, MA, USA) and pUC-57 (Genescript, Piscataway, NJ, USA) plasmids (listed in **Supplementary Table S5)**, were cloned into the pCTCON plasmid using the following protocol. NheI and BamHI restriction sites were added to the N- and C-terminal ends of the APPI sequences by PCR amplification using the primers listed in **Supplementary Table S6** (Integrated DNA Technologies). The PCR products and the pCTCON plasmid were digested with NheI-HF and BamHI-HF restriction enzymes (New England Biolabs). After digestion, calf intestinal phosphatase (CIP) (New England Biolabs) was added to the pCTCON plasmid to prevent self-ligation of the plasmid. The digested pCTCON vector was confirmed by running the vector on a 1% agarose gel, followed by DNA extraction with a HiYield™ Gel/PCR DNA Fragments Extraction Kit (RBC Bioscience, New Taipei City, Taiwan).

The digested APPI insert was then ligated into the digested pCTCON vector using T4 ligase (New England Biolabs). The ligation products were transformed into DH5α *Escherichia coli* cells to increase plasmid yield. The plasmid was purified with a HiYield Plasmid Mini Kit (RBC Bioscience), and the substituted positions and mutations were confirmed by Sanger sequencing of the extracted DNA [DNA Microarray and Sequencing Unit (DMSU), the National Institute of Biotechnology in the Negev (NIBN), BGU, Beer-Sheva, Israel].

**Construction and cloning of APPI variants in pPIC9k plasmid for recombinant protein production and purification**

The APPI variants were constructed by using one of two methods. The first method was based on site-directed mutagenesis, starting with the human cDNA of the APPI-3M template, previously cloned into the *Pichia* *pastoris* expression vector pPIC9K and later using APPI_T11R_ and APPI_P13L_ as cDNA templates. Site-directed mutagenesis was performed by amplifying these DNA templates via PCR using Phusion DNA polymerase and the mutagenesis primers listed in **Supplementary Table S5** (Integrated DNA Technologies). The resulting constructs included four restriction sites and two epitope tags (FLAG and HIS×6) in the following sequence: SnaBI-FLAG–EcoRI-APPI–AvrII-HIS×6–NotI. The PCR products were phosphorylated using T4 Polynucleotide Kinase and ligated with T4 ligase (New England Biolabs). The second method employed restriction-ligation cloning, where APPI variants (namely, APPI_T11K_,_P13I_ and APPI_G17Y, F18K_) from the pCTCON plasmid were cloned into the pPIC9K plasmid by adding EcoRI and AvrII restriction sites to the APPI gene sequence through PCR amplification, using primers listed in **Supplementary Table S7** (Integrated DNA Technologies). PCR products and the pPIC9K plasmid were digested with EcoRI-HF and AvrII-HF restriction enzymes (New England Biolabs). After digestion, CIP was added to the digested pPIC9K plasmid. The digestion of the plasmid was confirmed by running it on a 1% agarose gel, followed by DNA extraction from the gel using a HiYield™ Gel/PCR DNA Fragments Extraction Kit.

The APPI constructs were transformed into DH5α *E. coli* cells to produce large quantities of plasmid DNA for transformation in yeast. Mutations were confirmed by Sanger sequencing of the extracted DNA (DMSU, NIBN, BGU). DNA maxi-prep was performed according to the manufacturer’s instructions (MACHEREY-NAGEL, Düren, Germany). Approximately 100 μg of each plasmid was linearized with the restriction enzyme SacI-HF (New England Biolabs) and subsequently transformed into freshly prepared electrocompetent *P. pastoris* GS115 cells, following the pPIC9K protocol (Invitrogen). This process resulted in the insertion of the construct at the AOX1 (alcohol oxidase) locus of *P. pastoris*, thereby generating a His+ Mut+ phenotype and Geneticin resistance, since the plasmid encodes histidinol dehydrogenase and contains the bacterial kanamycin resistance gene. Transformants were selected for the His+ phenotype on 2% agar containing regeneration dextrose biotin (RDB; 18.6% sorbitol, 2% dextrose, 1.34% yeast nitrogen base, 4 × 10^−5^ % biotin, and 0.005% each of l-glutamic acid, l-methionine, l-lysine, l-leucine, and l-isoleucine) and allowed to grow for 2 days at 30°C. The cells were harvested from the plates and subjected to further selection for a high copy number by their ability to grow on 2% agar containing 1% yeast extract, 2% peptone, 2% dextrose medium, and 4 mg/ml Geneticin (Invitrogen). To verify the insertion of the construct at the AOX1 locus, genomic DNA from each APPI variant was extracted following an established protocol ^2^ and amplified using an AOX1 upstream primer 5′-GACTGGTTCCAATTGACAAGC-3′ and an AOX1 downstream primer 5′-GCAAATGGATTCTGACATCC-3′ (Integrated DNA Technologies). PCR products were analyzed on a 1% diagnostic agarose gel to confirm the correct product sizes. Two bands were observed, one corresponding to the alcohol oxidase gene (~2000 bp) and one to the APPI gene (~700 bp). The APPI DNA fragment was extracted from the agarose gel and sequenced to verify the accuracy of the gene constructs.

**YSD technique**

Human cDNA encoding APPI variants cloned into the pCTCON plasmid was transformed into freshly prepared competent *Saccharomyces cerevisiae* EBY100 cells by electroporation with a MicroPulser electroporator (Bio-Rad, CA, USA), according to the protocol established by Chao et al. ^3^. Transformed yeast cells were cultivated on SDCAA selective medium (15% agar, 2% dextrose, 1.47% sodium citrate, 0.429% citric acid monohydrate, 0.67% yeast nitrogen base, and 0.5% casamino acids) with a pCTCON vector providing tryptophan-based selection. For induction of protein expression, colonies were transferred to SGCAA medium, which is the same as SDCAA but with 2% galactose instead of dextrose to activate the GAL promoter. Surface display of APPI variants on the yeast in the presence of fluorescently labeled proteases is illustrated in **Supplementary Figure S1A**. Yeast cells were incubated at 4°C for 1 h with 70 nM KLK6 labeled with allophycocyanin (APC) and 250 nM biotinylated (EZ-Link™ NHS-PEG4 Biotinylation Kit, ThermoFisher Scientific, Waltham, MA, USA) catalytically inactive mesotrypsin-S195A ^4,5^, followed by a 30-min incubation with a 1:800 dilution of fluorescein isothiocyanate (FITC)-conjugated streptavidin (ThermoFisher Scientific). For preparing APC-labeled KLK6, recombinant pro-KLK6 (comprising the stabilizing mutations R74G, R76Q, and N132Q) was expressed and purified from a virus/insect cell line system Sf21 (Protein Expression and Purification Core Facility, EMBL Heidelberg, Germany) ^6^ and then labeled with APC (ThermoFisher Scientific), following the manufacturer’s protocol, at a ratio of 1:5 (KLK6/APC). To accommodate intermediate-affinity variants, protease concentrations were increased 10-fold compared to those used for the DMS study (25 nM for mesotrypsin and 7 nM for KLK6) ^7^. In the DMS study, those concentrations had been optimized to provide an equivalent distribution of staining intensities across the yeast-displayed variants, but in the current study they did not provide sufficient sensitivity to detect weak binders. By increasing the concentrations of the protease – to 250 nM for mesotrypsin and 70 nM for KLK6 – the interaction between the protease and the intermediate-affinity variants was enhanced, thereby producing stronger fluorescence signals. This adjustment improved our ability to measure binding events across a broader range of affinities, including those of weak APPI binders. After incubation of the yeast cells with the relevant protease, the cells were washed with PBSA buffer (137 mM NaCl, 2.7 mM KCl, 10 mM Na_2_HPO_4_, 1.8 mM KH_2_PO_4_, and 1% BSA). Thereafter, fluorescence intensities of the FITC- (490 nm) and APC- (650 nm) conjugates were determined using a BD FACS Canto II flow cytometer (BD Biosciences, Belgium) (**Supplementary Figure S1B**). The higher the fluorescence signal, the stronger the binding affinity of the variant to the respective protease.

Concurrently with monitoring affinity of the YSD APPI variants to mesotrypsin and KLK6, the expression levels of those variants were quantified by treating the yeast cells with a 1:50 dilution of 9E10 mouse anti-c-Myc antibody (Abcam, Cambridge, UK), followed by a phycoerythrin (PE)-conjugated secondary antibody (Abcam) (**Supplementary Figure S1C**). Variants with high surface expression yielded an intensified fluorescence signal at 565 nm, corresponding to the emission wavelength of PE.

**Protein production and purification**

Transformed yeast cells were induced for small-scale production to identify colonies with the highest expression yield of each APPI variant. Multiple colonies for each APPI variant were grown overnight at 30°C with shaking at 300 rpm in 5 ml of BMGY medium (2% peptone, 1% yeast extract, 0.23% K₂HPO₄, 1.18% KH₂PO₄, 1.34% yeast nitrogen base, 4×10⁻⁵ % biotin, 1% glycerol). Expression was induced by growing the cells for three days in 5 ml of BMMY medium (same as BMGY, but with 0.5% methanol instead of glycerol), while adding 0.5% methanol daily. After three days, cells were precipitated by centrifugation at 4000 rpm for 5 min in order to separate out the medium containing the secreted APPI protein variants. Expression of APPI variants, each one from several colonies, was determined in terms of their ability to inhibit the catalytic activity of bovine trypsin (Sigma, Saint Louis, MO). In that assay, 5 nM bovine trypsin in TB buffer (100 mM Tris, 1 mM CaCl₂, pH 8.0) was mixed with 5 µl of supernatant from each colony expressing the APPI variant. Upon addition of the substrate Z-GPR-pNA to a final concentration of 75 µM, reactions were monitored spectroscopically for 5 min using a Synergy2 microplate spectrophotometer (BioTek, Winooski, VT, USA). Initial rates of substrate cleavage were determined by the increase in absorbance at 410 nm caused by the cleavage of Z-GPR-pNA and the release of p-nitroaniline (pNA). The inhibitory effect of the APPI variant in the supernatant reflects the amount of APPI produced by each yeast colony. The colony with the most potent inhibitory effect gave the highest expression yield and was therefore selected for further large-scale production of the APPI variant.

The cells with the highest expression of each APPI variant were first transferred into 50 ml of BMGY and grown to an *A*_600_ = 10.0 (10^8^ cells/ml), followed by scaling-up to 500 ml of BMGY, until an *A*_600_ = 10.0 was reached (by an overnight growth at 30°C with shaking at 300 rpm). The cells were harvested by centrifugation, resuspended in 1 l of BMMY, and grown at 30°C with shaking at 300 rpm to *A*_600_ = 5.0 to induce expression. Methanol was added to a final concentration of 2% every 24 h to maintain induction. Following five days of induction, the culture was centrifuged, and the supernatant, containing the relevant secreted APPI variant, was prepared for purification by nickel-immobilized metal affinity chromatography by using the C-terminal HIS tag in each variant. The supernatant was filtered through a 0.22-μm Stericup bottle-top filter (Millipore, Billerica, MA, USA). The filtered supernatant was adjusted to 10 mM imidazole and 0.5 M NaCl at pH 8.0 and incubated for 1 h at 4°C. Then, a second filtration step was performed to remove any additional precipitates. The resulting supernatant was loaded onto a HisTrap 5-ml column (GE Healthcare, UK) at a flow rate of 0.7 ml/min for 24 h, washed with a washing buffer (20 mM sodium phosphate, 0.5 M NaCl, and 10 mM imidazole; pH 8.0), and eluted with an elution buffer (similar to the washing buffer, but with 0.5 M imidazole) in an ÄKTA pure instrument (GE Healthcare).

Gel-filtration chromatography was performed with a Superdex 75 16/600 column (GE Healthcare) equilibrated with PBS buffer (137 mM NaCl, 2.7 mM KCl, 8 mM Na_2_HPO_4_, and 2 mM KH_2_PO_4_, pH 7.4), at a flow rate of 1 ml/min on an ÄKTA start instrument. SDS-PAGE analysis on a 15% polyacrylamide gel under reducing conditions was used to confirm the purity of the proteins. The correct mass of the pure proteins was validated using a MALDI-TOF REFLEX-IV (Bruker, Billerica, MA, USA) mass spectrometer (The Ilse Katz Institute for Nanoscale Science and Technology, BGU). Purification yields for all APPI clones were determined using a NanoDrop spectrophotometer (Thermo Scientific), based on absorbance at 280 nm, with the corresponding extinction coefficient of each variant calculated using Expasy ProtParam. Since the NanoDrop measures the total concentration of the inhibitor and not the active inhibitor concentration, an activity-based titration assay was also performed, using bovine trypsin, following an established protocol ^8^ to determine the active amount of inhibitor used in the inhibition assays. Bovine trypsin was mixed with a range of substoichiometric concentrations of APPI and then assayed for residual activity with l-BAPA (100 µM, Nα-benzoyl-l-arginine 4-nitroanilide; Sigma, Saint Louis, MO, USA) as the substrate. A plot of residual enzyme activity vs. APPI concentration allowed extrapolation to the stoichiometric equivalence point. This extrapolation was justified as all APPI variants maintained consistently high affinity for bovine trypsin, binding to the enzyme in a 1:1 stoichiometric ratio.

**Inhibition studies**

The inhibition constants (*K*_i_) of the APPI variants for mesotrypsin and KLK6 were determined according to a previously described methodology with some modifications ^37^. For the experiment with classic inhibition of mesotrypsin, the substrate was Z-GPR-pNA, used at concentrations of 10, 25, 50, 100, and 250 µM; the inhibitor concentrations ranged between 0 nM to 3000 nM (depending on the variant); and the protease concentration was 0.25 nM. For the experiment with the tight binding model, the substrate was used at only one concentration of 150 µM; the inhibitor concentrations ranged between 0 to 10 nM; and the protease concentration was 25 pM. The dilution buffers included an inhibitor dilution buffer (10 mM Tris, pH 8.0, 0.1 mg/ml BSA), an enzyme dilution buffer (20 mM NaCl, pH 4.5, 1 mg/ml BSA, 10 mM CaCl₂), and a binding buffer (100 mM Tris, pH 8.0, 1 mM CaCl₂, 1% BSA). The reaction was initiated after incubation of the components for 10 min at 37°C, and the protease was added into a non-binding, 96-well microplate (Greiner, Kremsmünster, Austria) containing a mixture of substrate and inhibitor in binding buffer. The reaction was followed spectroscopically for 5 min for the classic inhibition assay and 3 h for the tight binding assay, using a Synergy2 microplate spectrophotometer (BioTek). Initial rates were determined from the increase in absorbance (410 nm) caused by the release of pNA upon mesotrypsin cleavage.

For the experiments with KLK6, the following conditions were employed: the substrate BOC-FSR-AMC was used at five concentrations, namely 250, 500, 1000, and 2000 µM. The concentration of the inhibitor ranged between 0 nM and 30,000 nM (depending on the variant), and the concentration of the protease was 1 nM. The dilution buffer was a KLK6 assay buffer (50 mM Tris, pH 7.3, 100 mM NaCl, 0.2% BSA). The reaction was initiated after incubating the components for 1 h at 37°C, after which time a mixture of the protease and the inhibitor was added to a black non-binding, 96-well microplate (Greiner) containing the above five concentrations of substrate. The fluorescent signal of the reactions was monitored for 5 h at 355 nm for excitation and 460 nm for emission.

**Supplementary Results**

**Production and purification of APPI variants**

The APPI variants were produced with the *P. pastoris* expression system and engineered with a C-terminal histidine tag for purification. The variants were purified using nickel-affinity chromatography (**Supplementary Figure S4A**), followed by size-exclusion chromatography (SEC) (**Supplementary Figure S4B**). All variants demonstrated high expression and purity and were produced with the correct molecular weight, as confirmed by SDS-PAGE analysis (**Supplementary Figures S4C, D**) and mass spectrometry (**Supplementary Figure S5**).

Following protein purification, the *K*_i_ values for mesotrypsin or KLK6 inhibition by APPI variants were determined by using data obtained from catalytic inhibition assays and fitted by multiple regression to the classic competitive inhibition equation (equation 7) or the Morrison tight-binding equation (equation 8) (**Supplementary Figures S6** and **S7**).

The selectivity index_inhibition_ for these variants was calculated on the basis of the logarithmic ratio of the *K*_i_ value for a specific variant in complex with KLK6 to the *K*_i_ value for the same variant in complex with mesotrypsin, normalized to the corresponding *K*_i_ of APPI-3M to establish a reference point for comparison of selectivity across different purified variants (equation 9). We chose the APPI-3M as the reference because it was the purified protein variant that exhibited relatively moderate *K*_i_ values for both targets (*K*_i_ = 98 pM for mesotrypsin and *K*_i_ = 362 pM for KLK6) ^9^.

**Supplementary Figures**


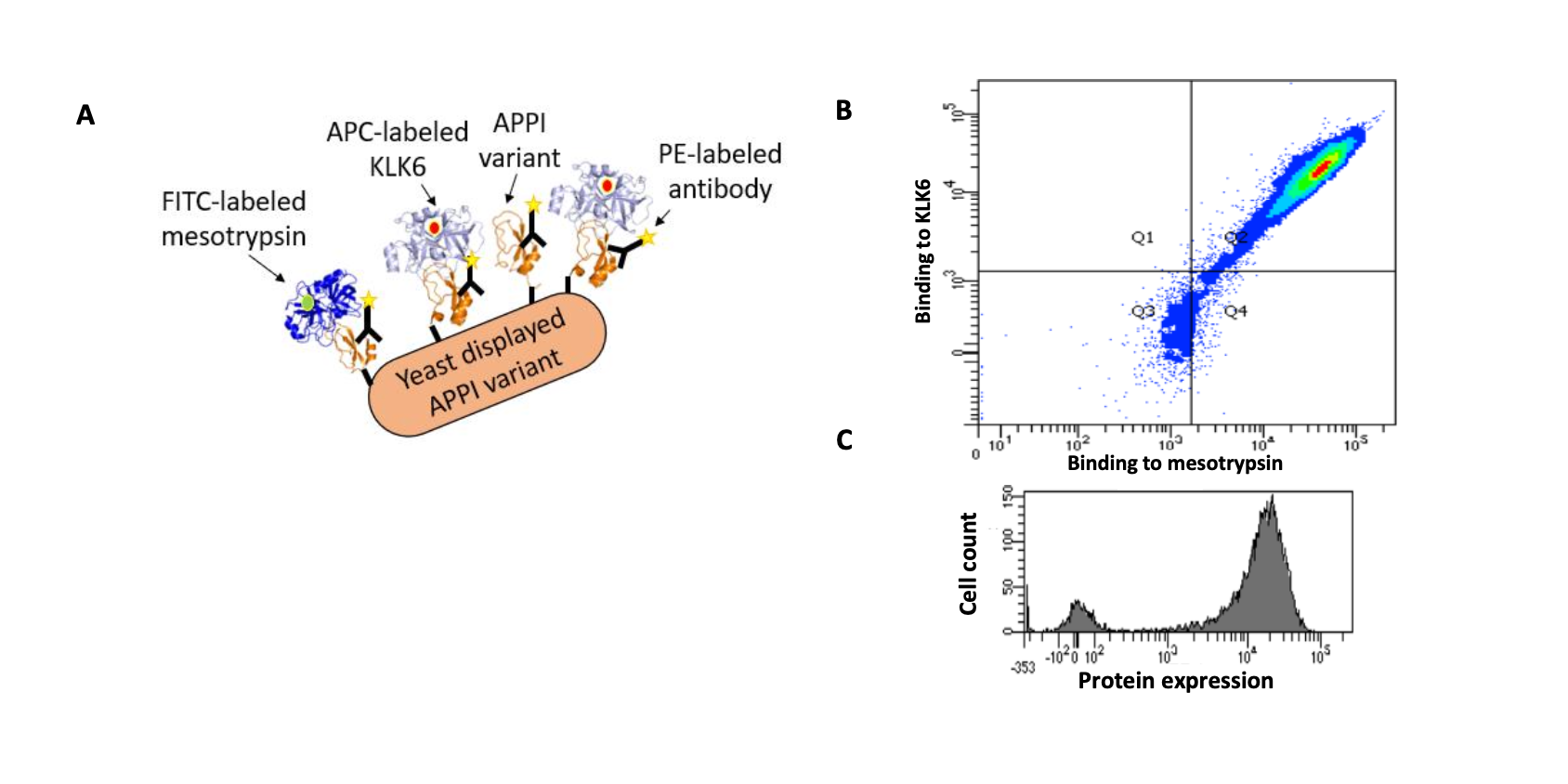


**Figure S1.** Selectivity determination using yeast surface display (YSD). (**A**) Schematic drawing of APPI variant binding measurements using the YSD system. The APPI variant was displayed on the yeast cell surface in the presence of labeled proteases, with mesotrypsin labeled with FITC and KLK6 labeled with APC. (**B**) Detection of variant binding to the two protease targets. (**C**) Determination of variant expression using a fluorescent anti-c-Myc antibody labeled with PE.


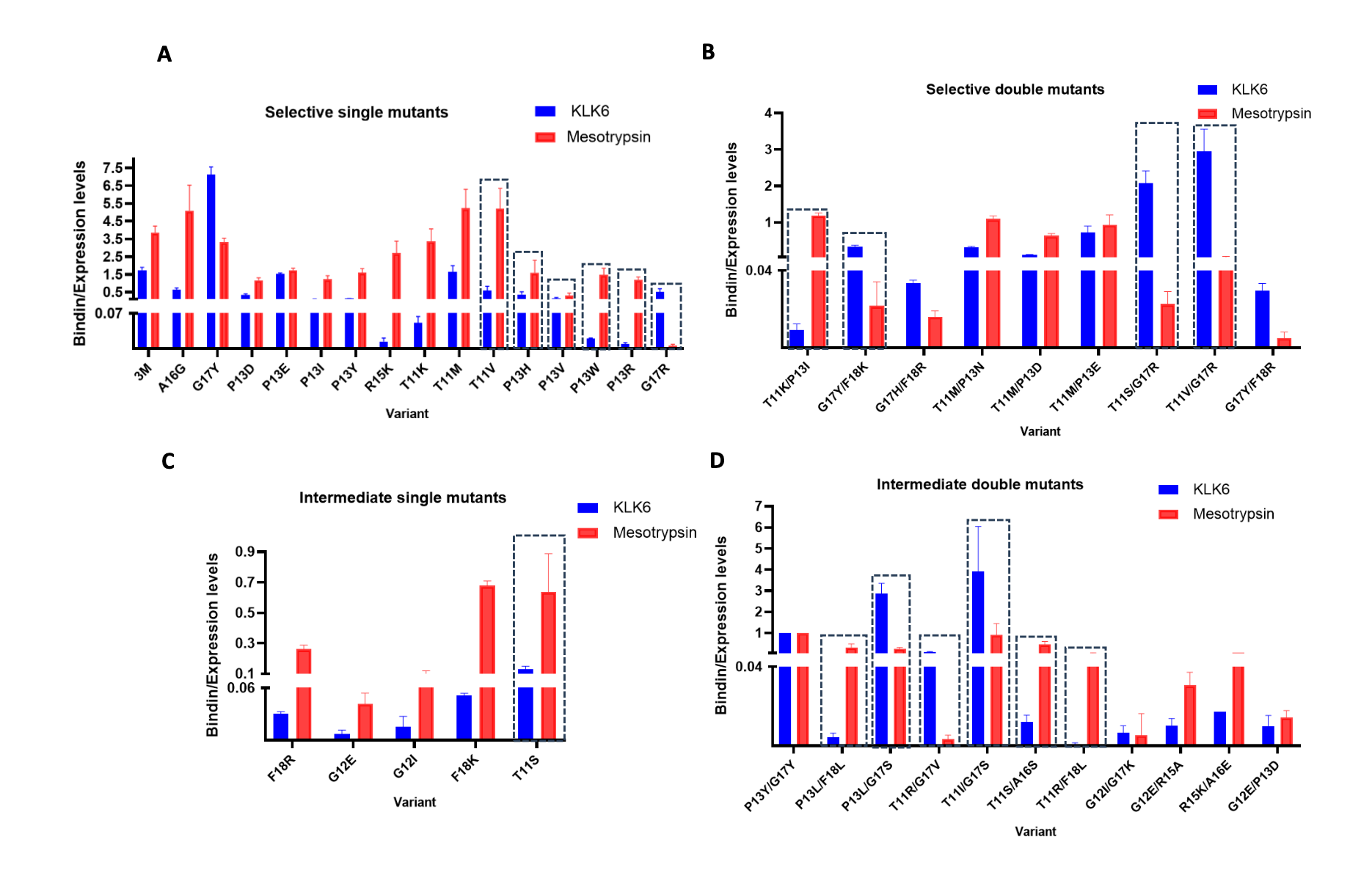


**Figure S2.** Binding determination using a YSD preliminary screen. Fluorescence intensity of variants in response to binding with fluorescently labeled proteases is shown. To prevent bias due to variable protein expression levels on the yeast surface, intensity values were normalized to the expression level of each variant (to obtain intrinsic binding values) and to the intrinsic binding of the reference variant APPI_P13Y,G17Y_, which exhibited moderate binding levels for both targets. Variants were grouped into four categories according to high or intermediate selectivity to mesotrypsin or KLK6, as determined by their predicted log_2_ selectivity ER values: (**A**) selective single mutants, (**B**) selective double mutants, (**C**) intermediate single mutants, and (**D**) intermediate double mutants. Black dashed lines indicate variants whose selectivity as soluble proteins was assessed via inhibition assays.

**
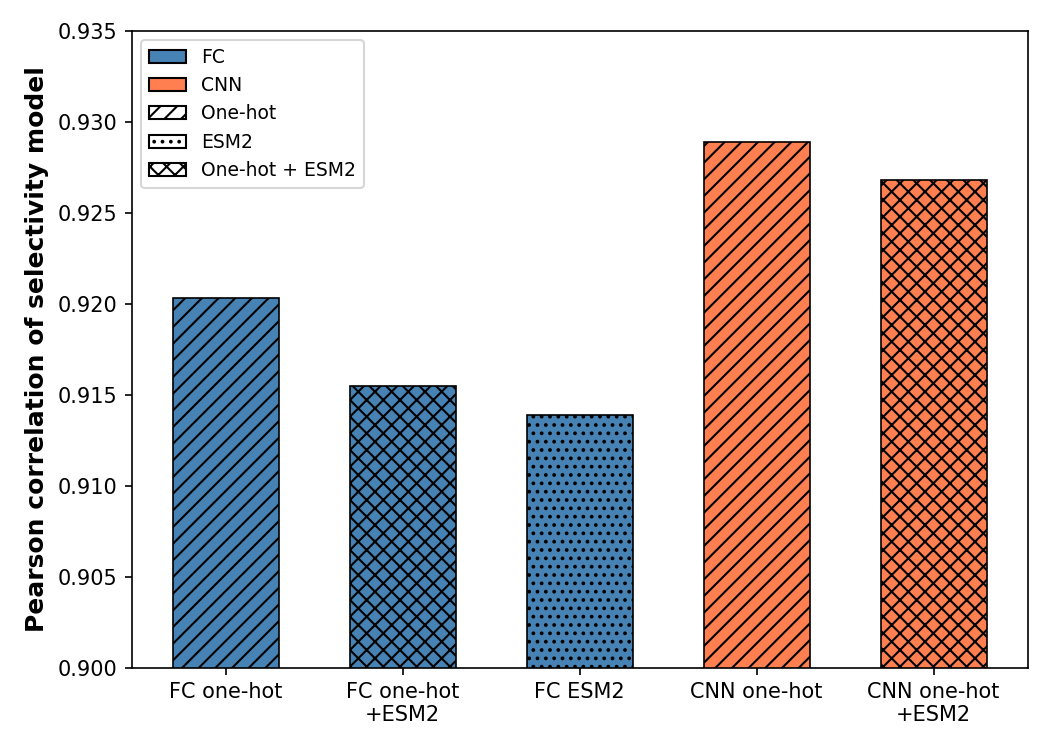
**

**Figure S3. Comparison of machine-learning models for selectivity prediction.**
Pearson correlation coefficients of the ${selectivity}_{model}$ on the validation set for five architectures: fully connected (FC) and convolutional-neural-network (CNN) models using different input representations (one-hot encoding, ESM2 embeddings, and their combination). We trained each model using hyper-parameters selected separately by random search for mesotrypsin and KLK6 prediction. **We report results on the held-out validation set of** 50 variants containing two mutations**.**


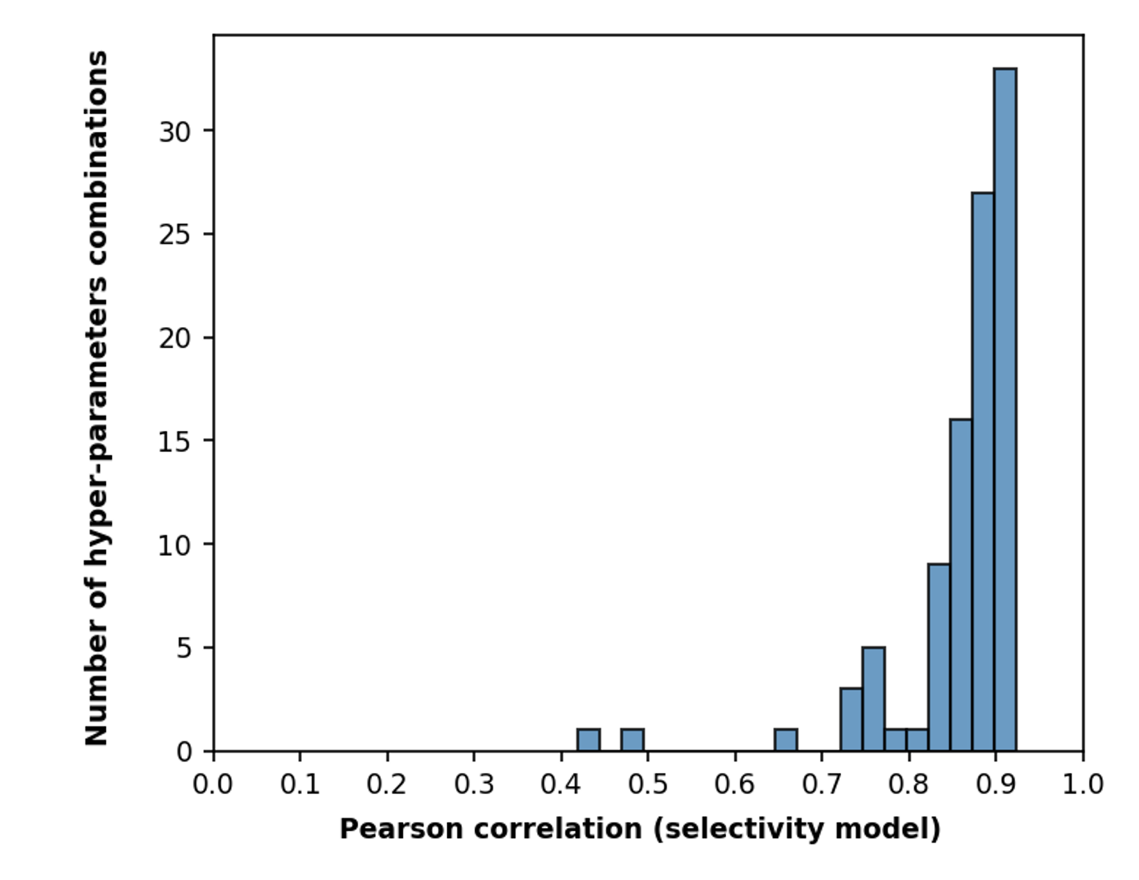


**Figure S4. Distribution of CNN model performance across hyper-parameter combinations.
Histogram of Pearson correlation values for the** ${selectivity}_{model}$ **obtained from 100 randomly sampled hyper-parameter combinations.**


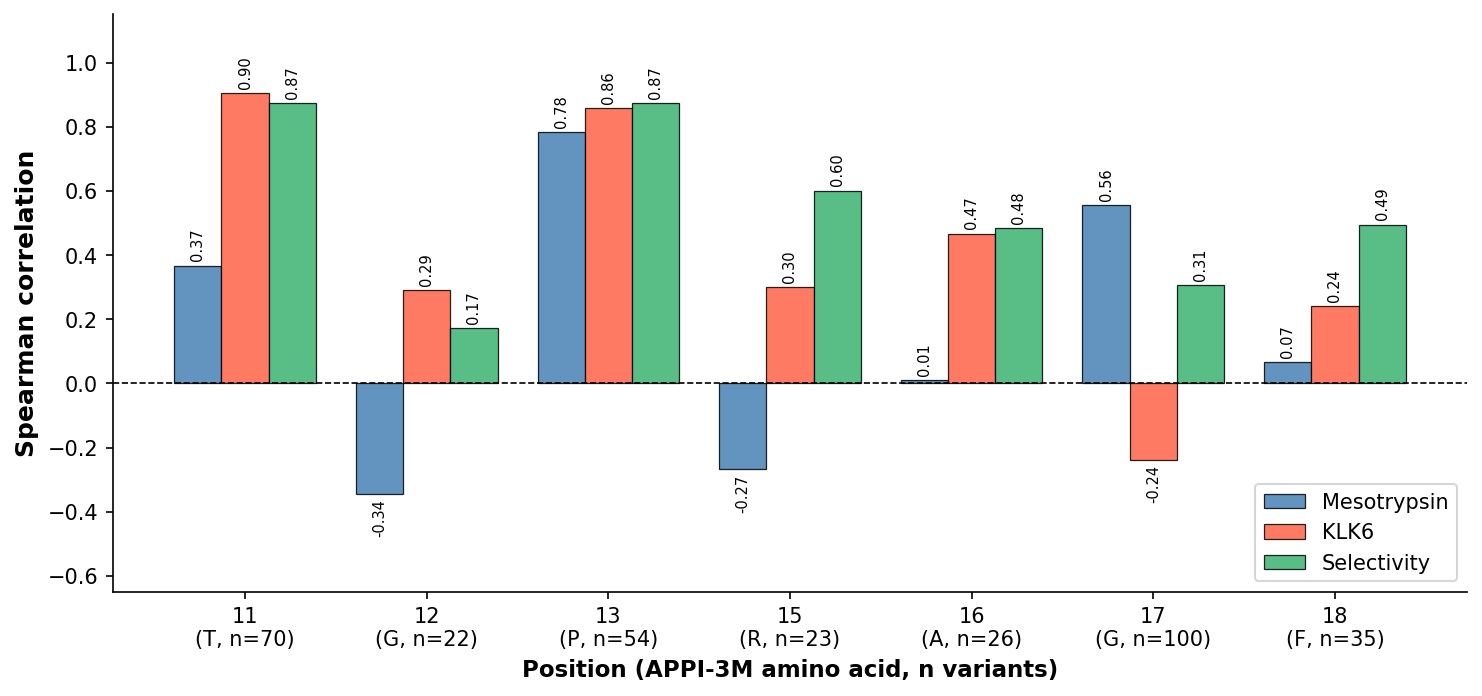

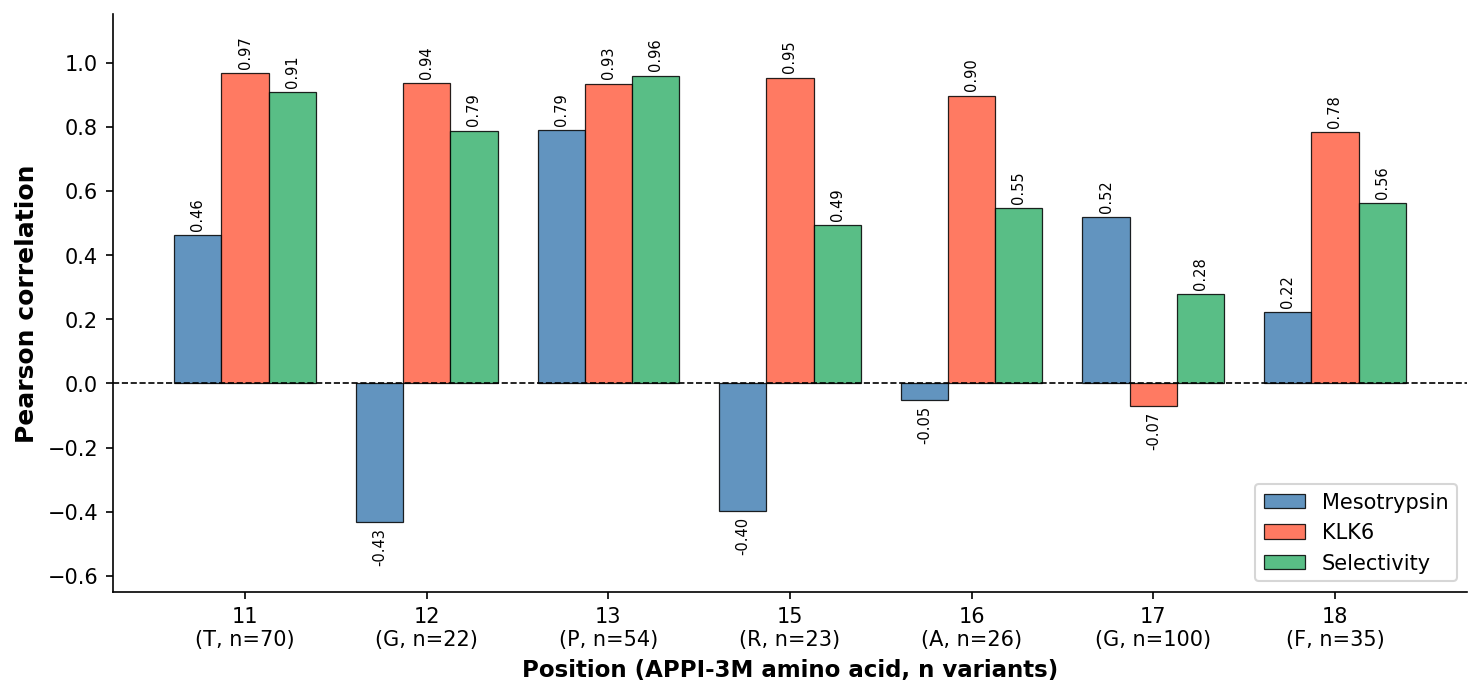


**Figure S5. Leave-one-position-out evaluation of model performance.** Bar plots show Pearson and Spearman correlation coefficients for ${mesotrypsin}_{model}$, ${KLK6}_{model}$, and ${selectivity}_{model}$ predictions when all variants containing mutations at a given position were excluded from training and used only for testing.

**
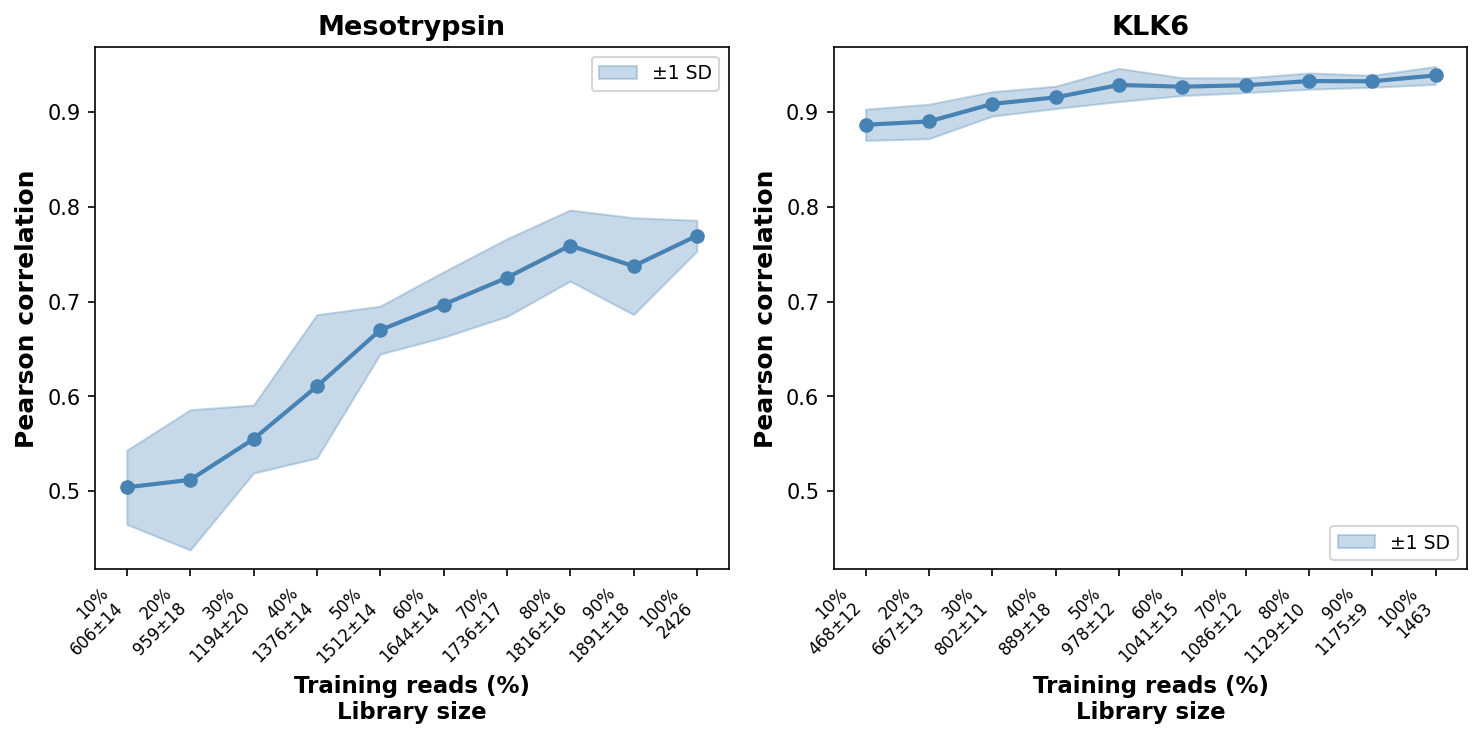
**

**Figure S6. Prediction performance as a function of the size of the sequencing depth. We report results on the held-out test set of** 49 variants containing two mutations**. A training set of 10%–100% of the rest of the data was randomly selected 10 times (excluding 100% of the data with a fixed training set). The library size of the training sets is shown as the mean ± standard deviation of 10 repeats.**

**
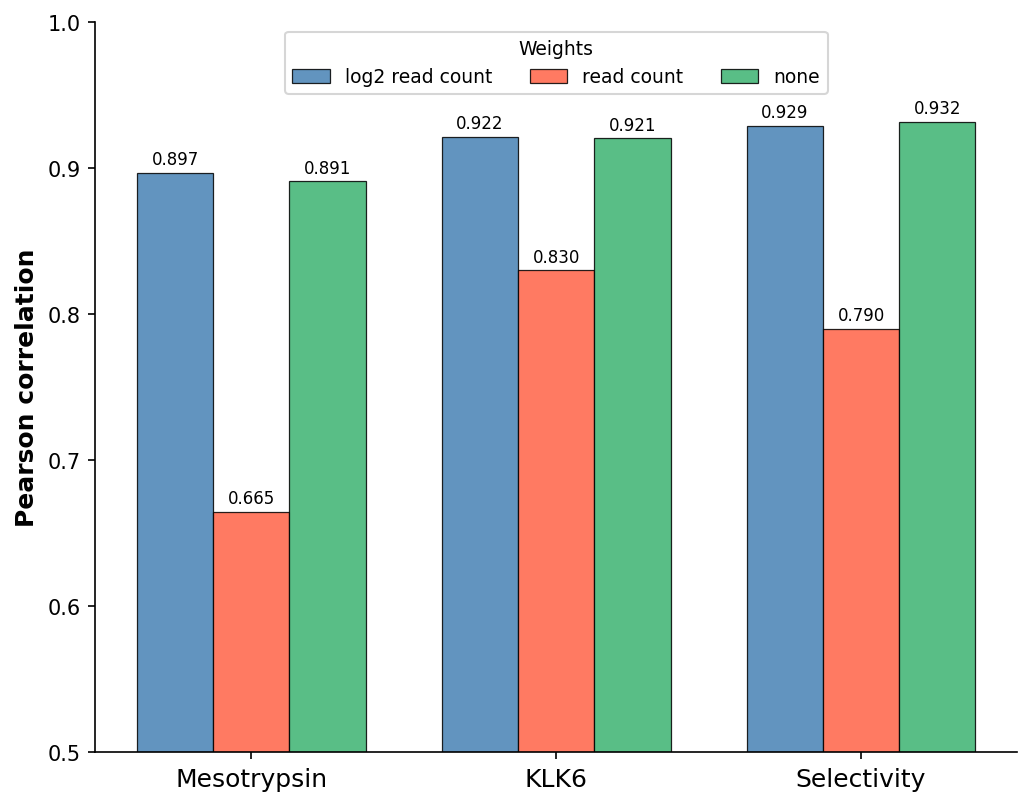
**

**Figure S7. Effect of sample weighting strategy on model performance.** Predictive performance (correlation between predicted and observed log2 ERs) of three sample weighting conditions: log2 read count, raw read count, and no weights. **We report results on the held-out validation set of** 50 variants containing two mutations**.**


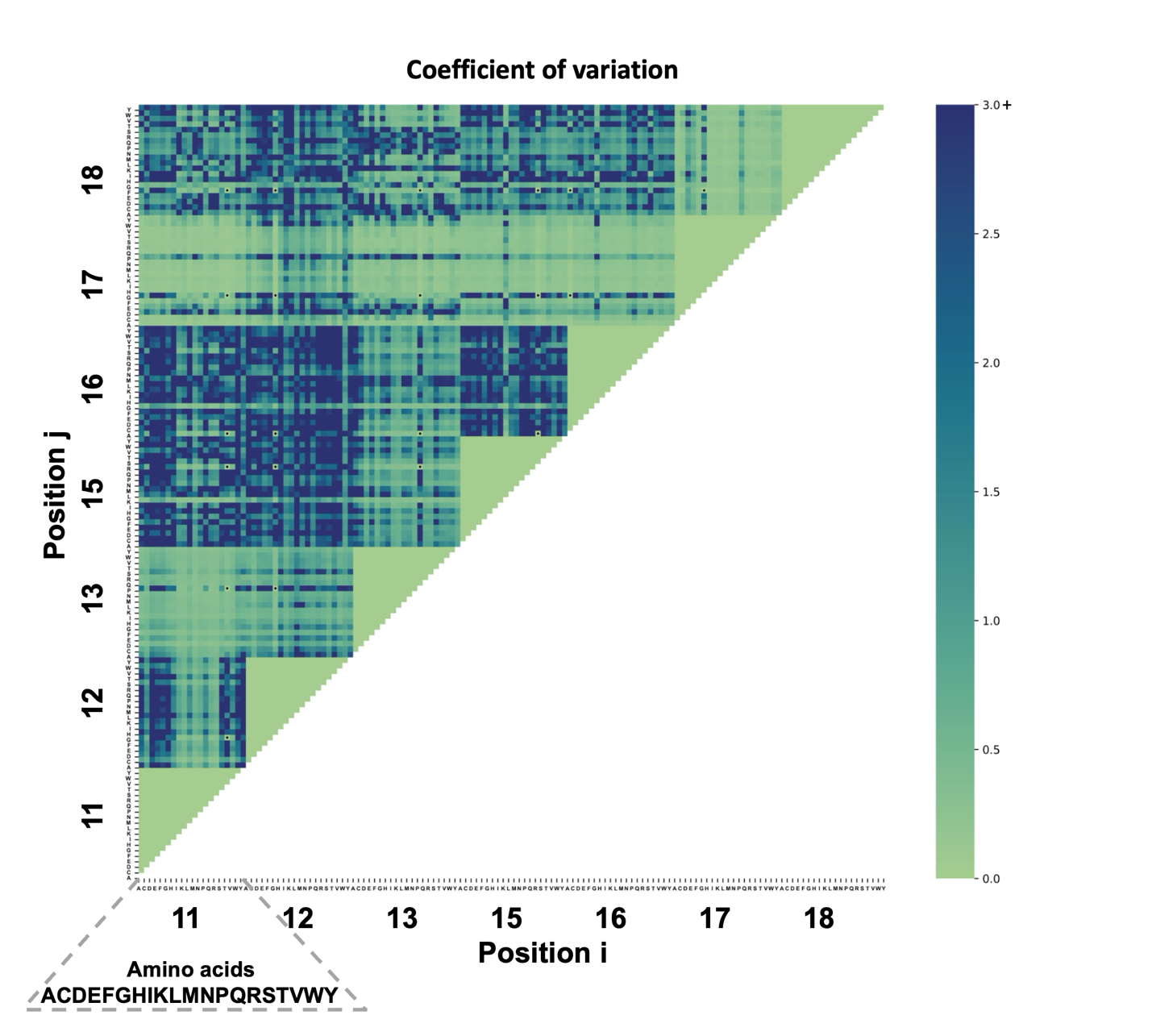


**Figure S8.** Heat map matrix of coefficients of variation for double mutations. Each square is a 20 × 20 matrix containing coefficient of variation values for each prediction of coupling between a mutation at position i with a mutation at position j.


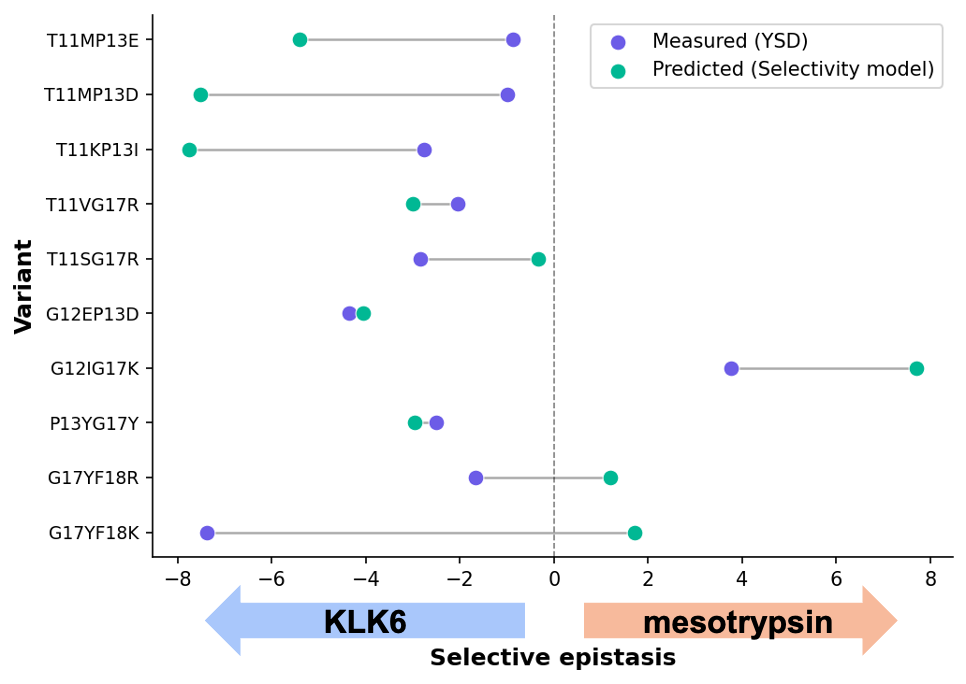


**Figure S9. Comparison of measured and predicted epistasis in double mutants. For each double mutant variant, experimentally measured selective epistasis (purple) is compared to** ${selectivity}_{model}$ **predicted selective epistasis (green), with connecting lines indicating the deviation between them. The dashed line at zero separates positive and negative selective epistasis.**


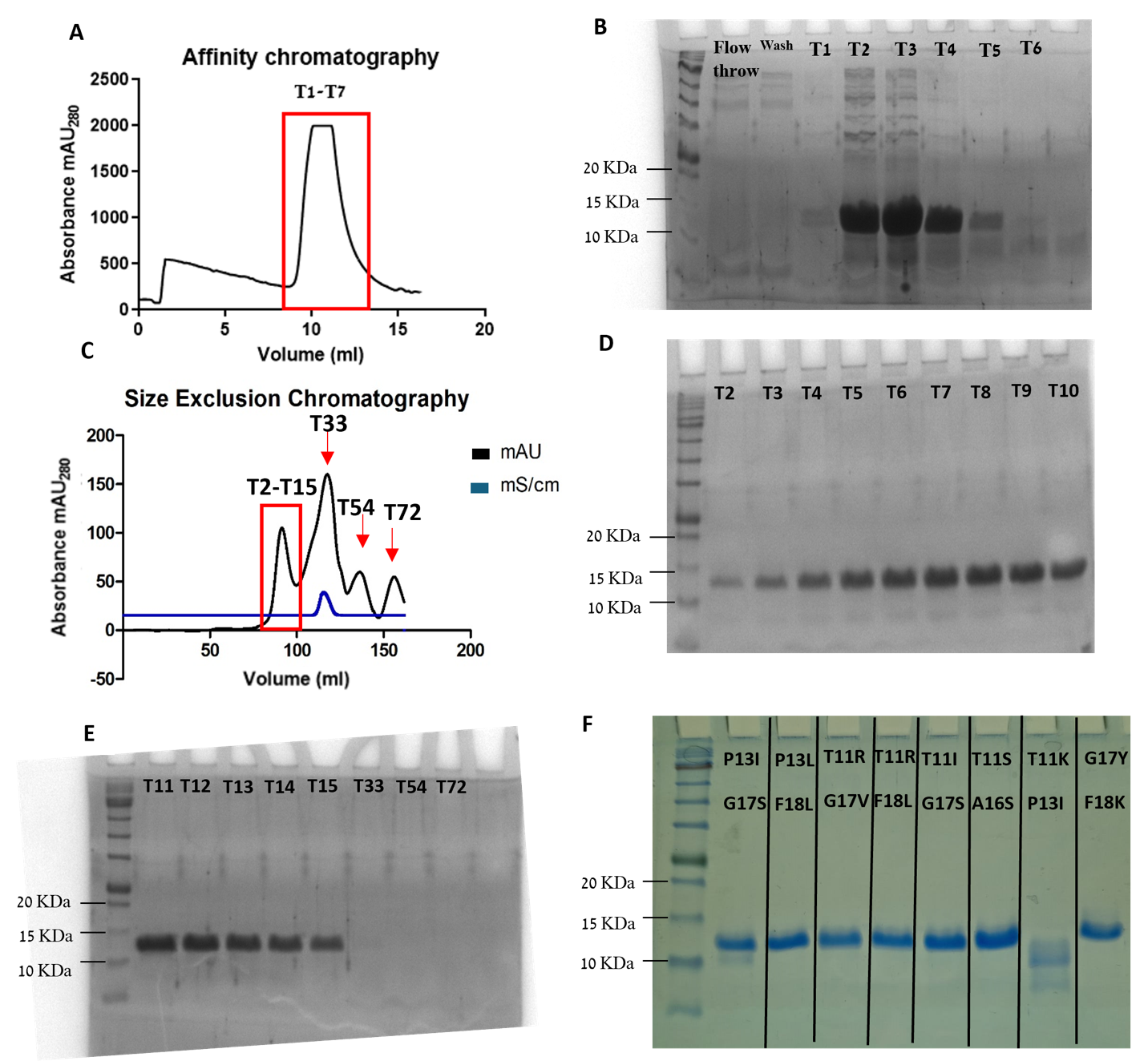


**Figure S10**. Purification process for the APPI variants. (**A–E**) Examples of the purification procedure for APPI_T11R,G17V_, where (**A**) nickel affinity chromatography purification profile; (**B**) SDS-PAGE analysis of fractions post affinity chromatography; (**C**) size-exclusion chromatography (SEC) profile, showing absorbance (black) and conductivity (blue) curves; and (**D,E**) SDS-PAGE analysis of fractions post SEC. (**F**) SDS PAGE of all the purified APPI variants. Variant APPI_T11K,P13I_ was designed such that it did not include a FLAG tag.


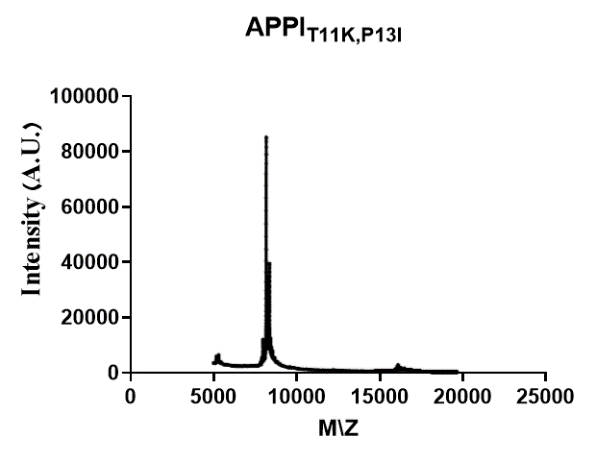

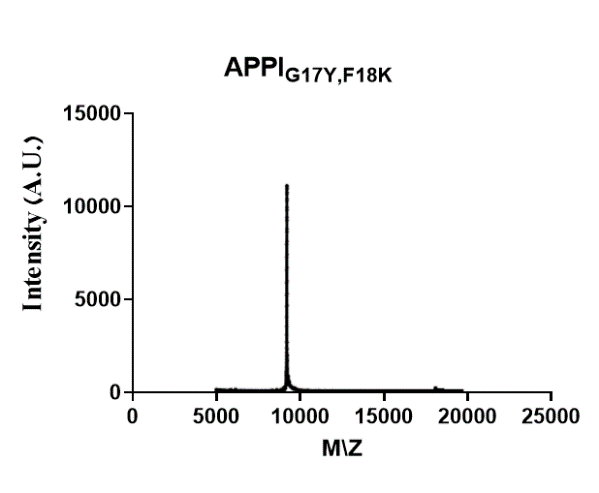

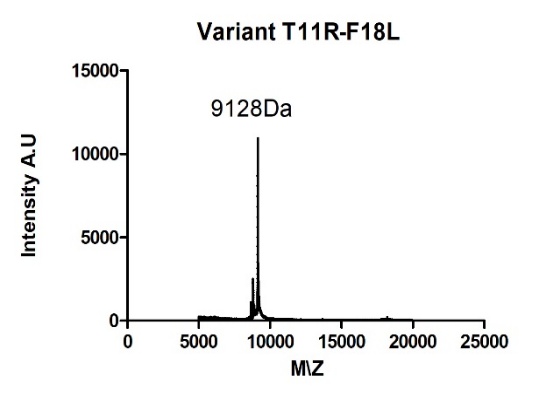

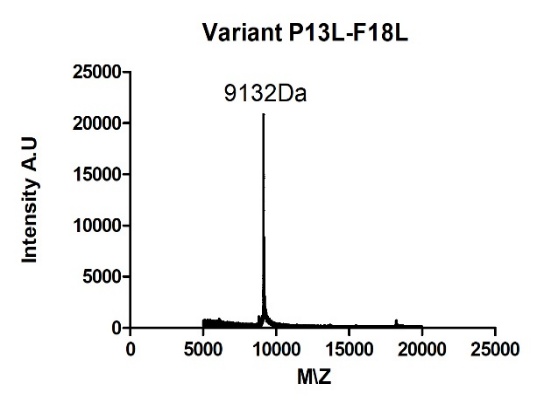

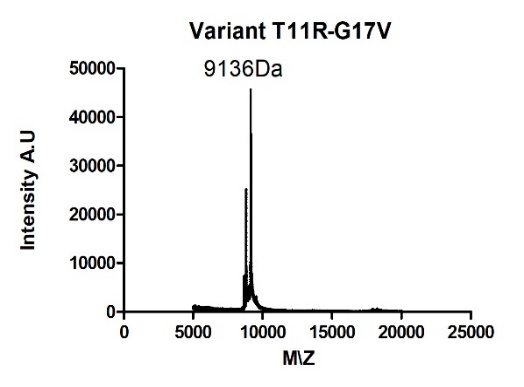

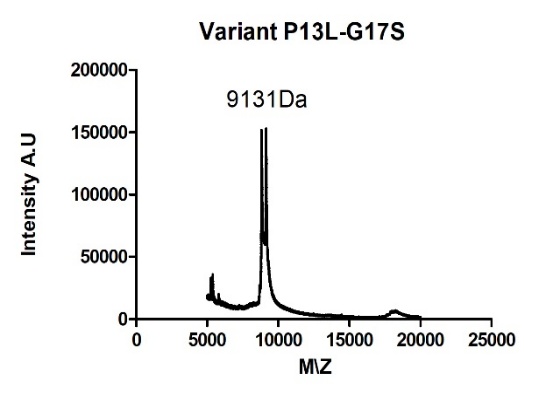


**A**

**B**

**C**

**D**

**Intensity (A.U.)**

**Intensity (A.U.)**

**Intensity (A.U.)**

**Intensity (A.U.)**

**Theoretical Mw: 9168 Da**

**Experimental Mw: 9131 Da**

**Theoretical Mw: 9123 Da**

**Experimental Mw: 9128 Da**

**Theoretical Mw: 9176 Da**

**Experimental Mw: 9136 Da**

**Theoretical Mw: 9104 Da**

**Experimental Mw: 9132 Da**


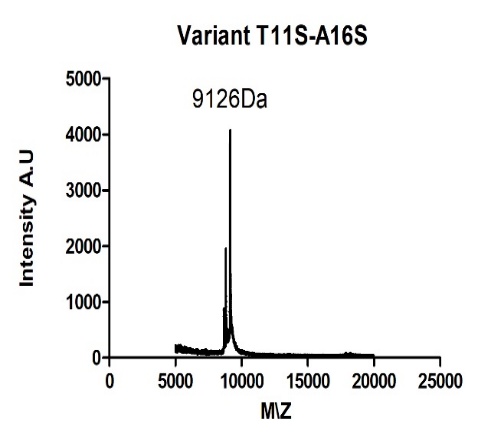

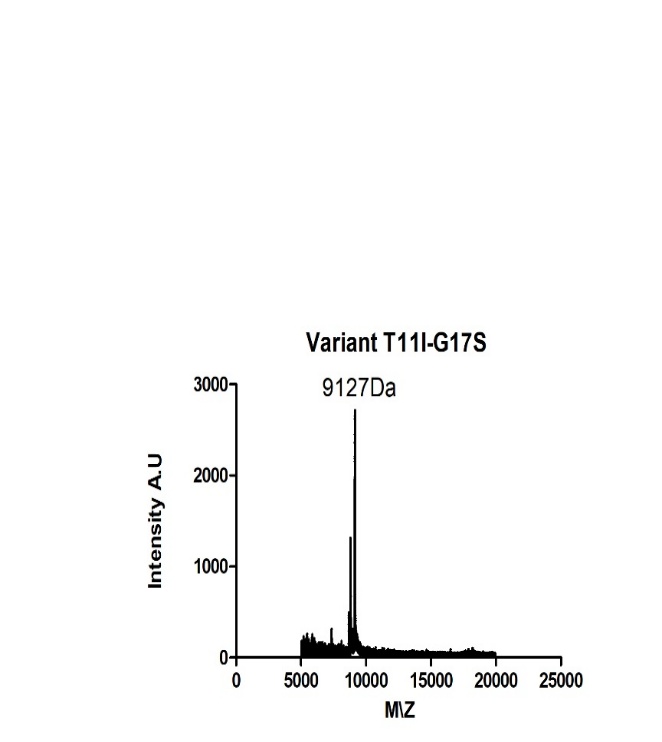


**G**

**E**

**Intensity (A.U.)**

**Intensity (A.U.)**

**Theoretical Mw: 9142 Da**

**Experimental Mw: 9127 Da**

**Theoretical Mw: 9163 Da**

**Experimental Mw: 9126 Da**

**Theoretical Mw: 8158 Da**

**Experimental Mw: 8155 Da**

**Theoretical Mw: 9209 Da**

**Experimental Mw: 9197 Da**

**F**

**H**


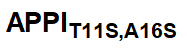

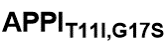

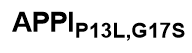

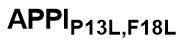

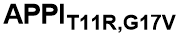

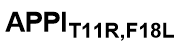


**Intensity (A.U.)**

**Intensity (A.U.)**

**Figure S11**. Mass spectrometry analysis of APPI variants. A.U. denotes Arbitrary Units, representing relative light intensity. The displayed peaks represent the average molecular weights (Mw) of each variant, given in daltons. The theoretical and experimental molecular weights of each APPI variant showed high similarity, confirming the correct molecular mass of the purified variant.


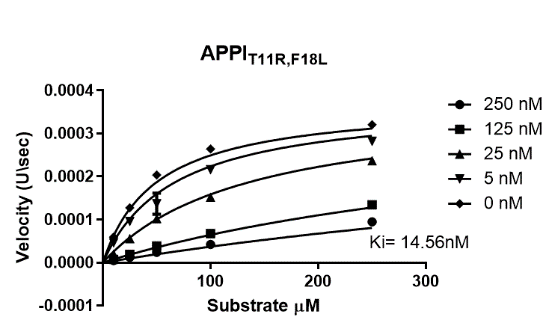

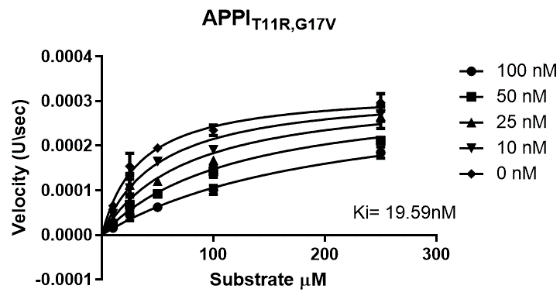

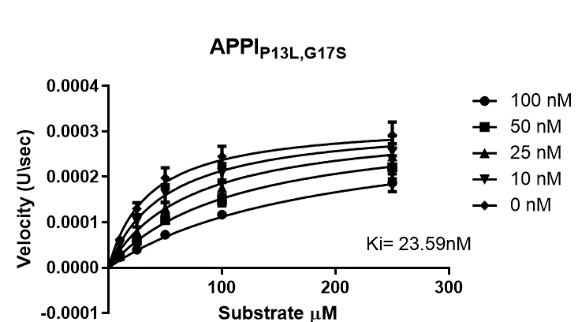

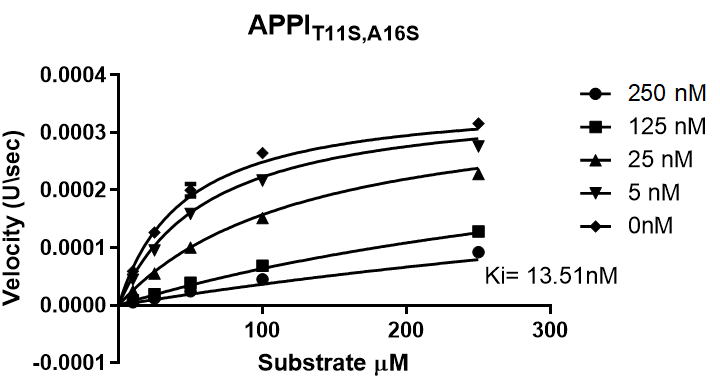

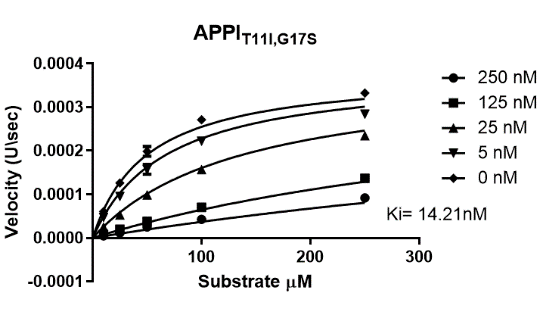


**A**

**D**

**C**

**B**

**E**

**F**

**G**

**H**


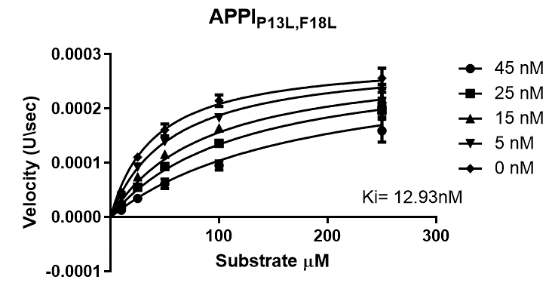

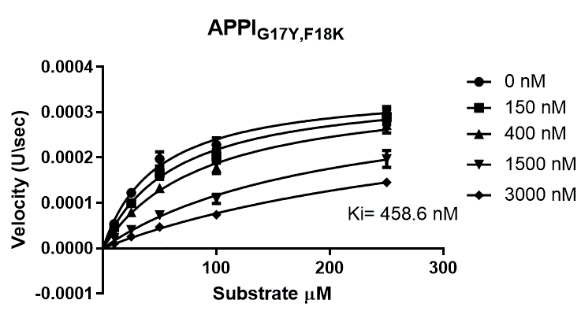

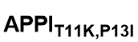


**APPI concentration (nM)**

**Substrate (**$\boldsymbol{\mu}$**M)**

**Substrate (**$\boldsymbol{\mu}$**M)**

**Substrate (**$\boldsymbol{\mu}$**M)**

**Substrate (**$\boldsymbol{\mu}$**M)**

**Substrate (**$\boldsymbol{\mu}$**M)**

**Substrate (**$\boldsymbol{\mu}$**M)**

**Substrate (**$\boldsymbol{\mu}$**M)**

**Velocity (**$\boldsymbol{s}^{\boldsymbol{-1}}$**)**

**Velocity (**$\boldsymbol{s}^{\boldsymbol{-1}}$**)**

**Velocity (**$\boldsymbol{s}^{\boldsymbol{-1}}$**)**

**Velocity (**$\boldsymbol{s}^{\boldsymbol{-1}}$**)**

**Velocity (**$\boldsymbol{s}^{\boldsymbol{-1}}$**)**

**Velocity (**$\boldsymbol{s}^{\boldsymbol{-1}}$**)**

**Velocity (**$\boldsymbol{s}^{\boldsymbol{-1}}$**)**

**Velocity (**$\boldsymbol{s}^{\boldsymbol{-1}}$**)**

**APPI_T11S,A16S_**

**APPI_P13L,G17Sv_**

**APPI_T11R,G17V_**

**APPI_T11I,G17S_**

**APPI_T11R,F18L_**

**APPI_P13L,F18L_**

**APPI_G17Y,F18K_**

**APPI_T11K,P13I_**

**Figure S12**. Kinetics of mesotrypsin inhibition by APPI. (**A-G**). Mesotrypsin cleavage of the peptide substrate Z-GPR-pNA is competitively inhibited by APPI variants. (H) The *K*_i_ for APPI_T11K,P13I_ was calculated using the tight-binding model (Morrison equation). The velocity of product formation at the beginning of the reaction was determined from the increase in absorbance (410 nm) caused by the release of pNA upon mesotrypsin cleavage.


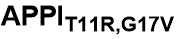

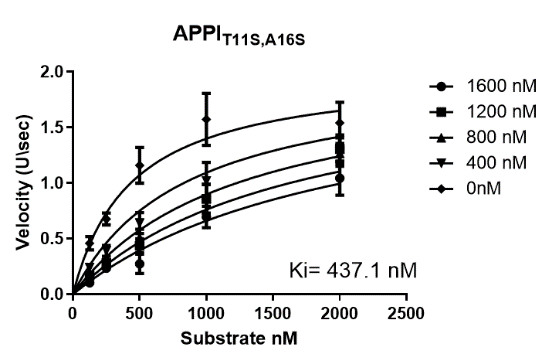

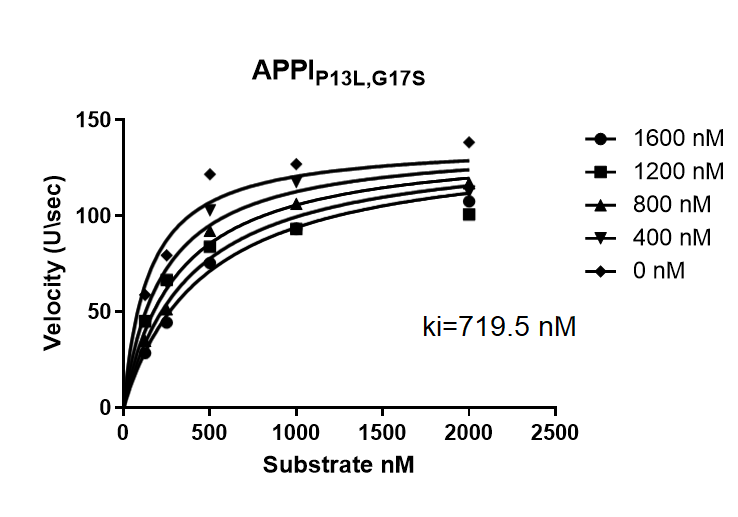

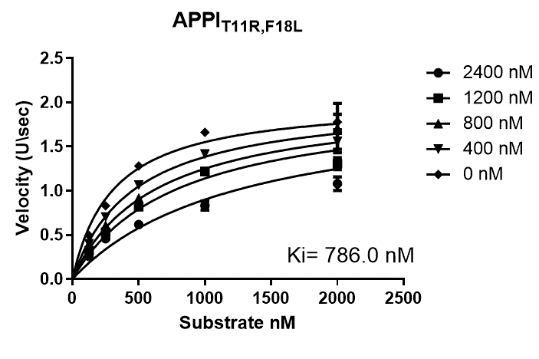


**G**

**H**


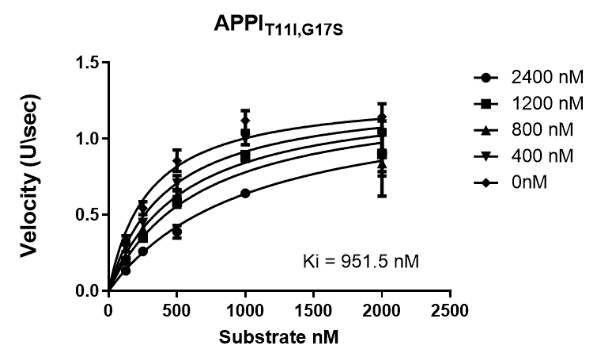

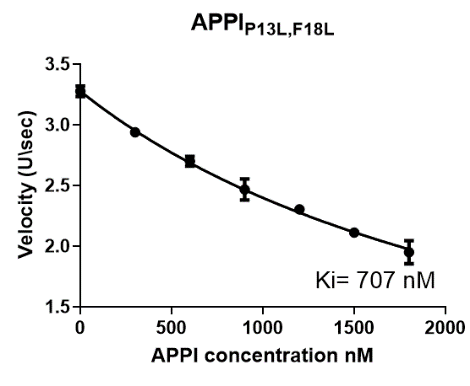

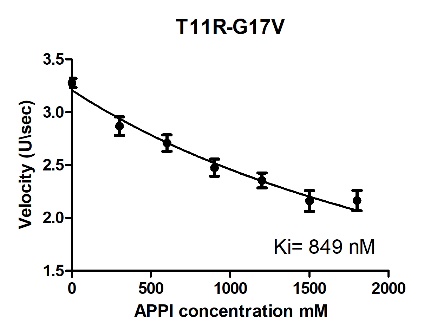


**A**

**BB**

**DB**

**EB**

**CB**


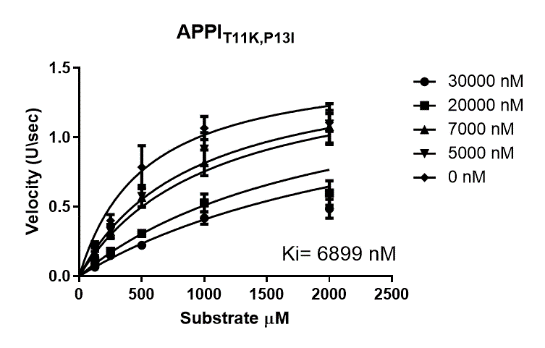

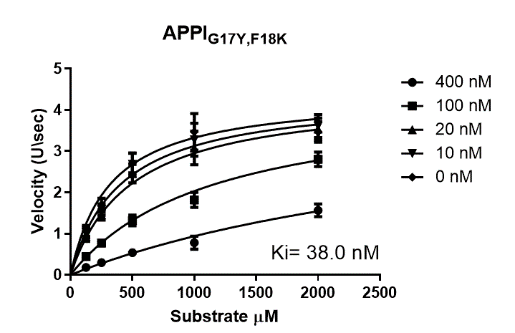


**F**

**APPI concentration (nM)**

**Substrate (**$\boldsymbol{\mu}$**M)**

**Substrate (**$\boldsymbol{\mu}$**M)**

**Substrate (**$\boldsymbol{\mu}$**M)**

**Substrate (**$\boldsymbol{\mu}$**M)**

**Substrate (**$\boldsymbol{\mu}$**M)**

**Substrate (**$\boldsymbol{\mu}$**M)**

**APPI concentration (nM)**

**Velocity (**$\boldsymbol{s}^{\boldsymbol{-1}}$**)**

**Velocity (**$\boldsymbol{s}^{\boldsymbol{-1}}$**)**

**Velocity (**$\boldsymbol{s}^{\boldsymbol{-1}}$**)**

**Velocity (**$\boldsymbol{s}^{\boldsymbol{-1}}$**)**

**Velocity (**$\boldsymbol{s}^{\boldsymbol{-1}}$**)**

**Velocity (**$\boldsymbol{s}^{\boldsymbol{-1}}$**)**

**Velocity (**$\boldsymbol{s}^{\boldsymbol{-1}}$**)**

**Velocity (**$\boldsymbol{s}^{\boldsymbol{-1}}$**)**

**APPI_P13L,G17S_**

**APPI_T11S,A16S_**

**APPI_T11K,P13I_**

**APPI_T11R,F18L_**

**APPI_T11I,G17S_**

**APPI_G17Y,F18K_**

**APPI_T11R,G17V_**

**APPI_P13L,F18L_**

**Figure S13**. Kinetics of KLK6 inhibition by APPI. (**A–F**) KLK6 cleavage of peptide substrate Boc-FSR-AMC is competitively inhibited by APPI variants. (**G, H**) The *K*_i_ values for APPI_T11R,G17V_ and APPI_P13L,F18L_, respectively, were calculated using the tight-binding model (Morrison equation). The velocity of product formation at the start of the reaction was determined from the increase in fluorescent signal caused by the release of AMC upon KLK6 cleavage.

**
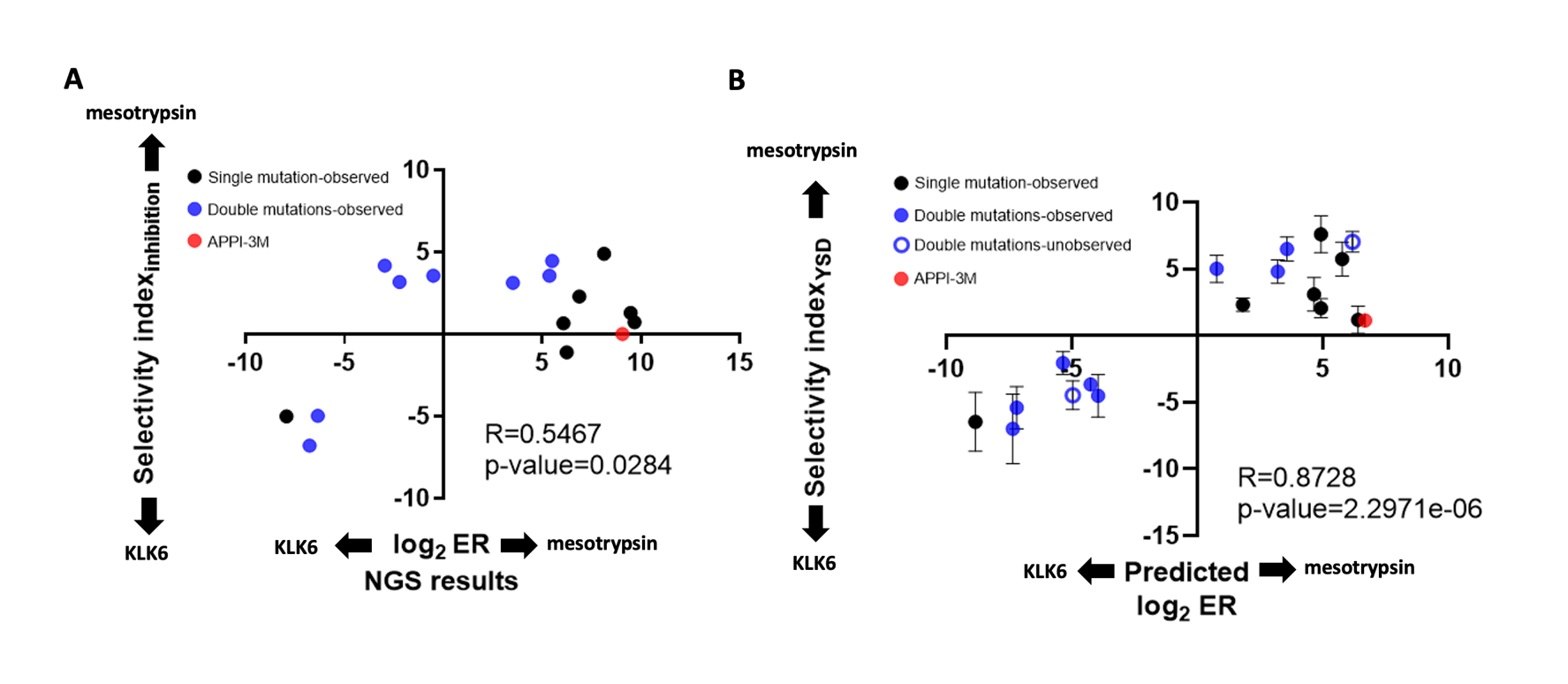
**

**Figure S14**. Performance of selectivity prediction. (**A**) Correlation between *K*_i_ constants ratio for selected variants and their selectivity log_2_ ER values as determined from DMS data. (**B**) Correlation of log_2_ selectivity ER, as predicted by the ${selectivity}_{model}$, with selectivity index_YSD_ being determined from fluorescence intensity binding signals using the YSD platform and FACS.

**Supplementary Tables**

**Table S1**. Search range for hyper-parameter values

| Hyper-parameter | Chosen value in $\boldsymbol{mesotrypsin}_{\boldsymbol{model}}$ | Chosen value in ${\boldsymbol{KLK}\boldsymbol{6}}_{\boldsymbol{model}}$ | Search range |
| --- | --- | --- | --- |
| Number of kernels | 8 | 8 | {4,8,16,32} |
| Kernel size | 5 | 5 | {2,3,4,5} |
| Pool size | 2 | 3 | {2,3,4,5} |
| FC layer 1 size | 64 | 4 | {4,8,16,32,64} |
| Dropout layer 1 | 0 | 0.1 | {0,0.1,0.2,0.3,0.4} |
| FC layer 2 | True | True | {True,False} |
| FC layer 2 size | 32 | 64 | {4,8,16,32,64} |
| Dropout layer 2 | 0 | 0 | {0,0.1,0.2,0.3,0.4} |
| Optimizer learning rate | 5×10^−4^ | 5×10^−3^ | {1×10^−4^, 5×10^−4^, 1×10^−3^, 5×10^−3^, 1×10^−2^, 5×10^−2^} |
| Batch size | 8 | 4 | {2,4,8,16,32,64} |
| Number of epochs | 50 | 50 | {10,20,30,40,50} |

**Table S2**. Selected APPI variants

| APPI variant | Predicted log_2_ selectivity ER | sd | CV | Crite­rion | Comment |
| --- | --- | --- | --- | --- | --- |
| 3M | 6.69 | 2.75 | 0.41 | N/A |  |
| A16G | 7.12 | 2.18 | 0.31 | N/A |  |
| F18R | -2.17 | 1.91 | 0.88 | N/A |  |
| G12E | -0.29 | 2.19 | 7.61 | N/A |  |
| G12EP13D | 2.85 | 2.07 | 0.73 | ii | Resulted in negative selective epistasis |
| G12ER15A | 1.60 | 2.12 | 1.33 | ii | Resulted in positive selective epistasis |
| G12IG17K | -0.72 | 1.51 | 2.10 | ii |  |
| G12I | -0.19 | 2.08 | 10.99 | N/A |  |
| G17K | -8.23 | 1.01 | 0.12 | N/A |  |
| G17Y | -5.15 | 1.24 | 0.24 | N/A |  |
| P13D | 7.19 | 2.12 | 0.30 | N/A |  |
| P13E | 3.30 | 2.46 | 0.74 | N/A |  |
| P13I | 7.20 | 2.44 | 0.34 | N/A |  |
| P13Y | 7.58 | 2.43 | 0.32 | N/A |  |
| R15K | 7.56 | 2.00 | 0.26 | N/A |  |
| R15KA16E | 2.97 | 2.14 | 0.72 | ii |  |
| T11K | 6.75 | 1.86 | 0.28 | N/A |  |
| T11M | 7.85 | 2.38 | 0.30 | N/A |  |
| T11KP13I | 6.19 | 1.87 | 0.30 | ii | Cold spots for mesotrypsin demonstrated negative epistasis for the mesotrypsin-to-KLK6 binding ratio when combined |
| T11MP13E | 5.73 | 2.26 | 0.39 | ii |  |
| T11MP13N | 7.94 | 2.21 | 0.28 | ii |  |
| T11MP13D | 7.52 | 1.98 | 0.26 | ii |  |

| P13LF18L | 3.57 | 1.87 | 0.52 | i | Cold spot for mesotrypsin combined cold spot for KLK6 |
| --- | --- | --- | --- | --- | --- |
| P13LG17S | -4.25 | 0.79 | 0.19 | i |  |
| T11RG17V | -3.95 | 0.83 | 0.21 | i |  |
| T11IG17S | -5.35 | 0.95 | 0.18 | i |  |
| T11RF18L | 3.20 | 1.96 | 0.61 | i |  |
| T11SG17R | -7.35 | 1.04 | 0.14 | i, ii |  |
| T11VG17R | -7.19 | 0.95 | 0.13 | i, ii |  |
| P13YG17Y | -0.54 | 1.53 | 2.86 | i |  |
| T11SA16S | 0.77 | 1.76 | 2.27 | ii |  |
| G17HF18R | -8.13 | 1.48 | 0.18 | ii | Cold spots for KLK6 showing positive selective epistasis for the mesotrypsin-to-KLK6 binding ratio when combined |
| G17YF18K | -4.96 | 1.73 | 0.35 | ii |  |
| G17YF18R | -6.13 | 1.46 | 0.24 | ii |  |
| F18K | -1.53 | 1.85 | 1.21 | N/A |  |
| T11V | 4.65 | 2.09 | 0.45 | N/A |  |
| P13V | 6.41 | 2.27 | 0.35 | N/A |  |
| P13W | 5.78 | 1.86 | 0.32 | N/A |  |
| P13H | 4.93 | 1.84 | 0.37 | N/A |  |
| P13R | 6.68 | 2.21 | 0.33 | N/A |  |
| T11S | 1.81 | 2.27 | 1.25 | N/A |  |
| G17R | -8.83 | 1.06 | 0.12 | N/A |  |

**Table S3**. Summary of selected variants kinetics, predicted log_2_ selectivity ER, and selectivity index (variants in bold are discussed in the main text)

| Variant | Predicted log2 selectivity ER | *K*_i_ against  KLK6 [nM] ^a^ | *K*_i_ against  mesotrypsin [nM] ^a^ | Selectivity index_inhibition_ (based on *K*_i_ values) | Selectivity toward target |
| --- | --- | --- | --- | --- | --- |
| P13L/F18L | 3.6 | 1051 ± 58 | 12.9 ± 1.4 | 4.5 | 81.5 |
| T11R/F18L | 3.2 | 786 ± 113 | 14.5 ± 1.1 | 3.9 | 54.2 |
| T11S/A16S | 0.8 | 437.1 ± 75.0 | 13.5 ± 0.8 | 3.1 | 32.4 |
| T11R/G17V | -4.0 | 708 ± 39 | 19.6 ± 3.0 | 3.3 | 36.1 |
| P13L/G17S | -4.3 | 719.5 ± 79.0 | 23.6 ± 4.0 | 3.2 | 30.5 |
| T11I/G17S | -5.3 | 951± 210 | 14.2 ± 0.9 | 4.2 | 67.0 |
| T11K/P13I | **6.2** | **1.8 ± 1.0** | **6899 ± 1240** | **10.0** | **3832.8** |
| G17Y/F18K | **-5.0** | **458.6 ± 46.0** | **38.0 ± 7.9** | **-5.5** | **12.1** |
| 3M^b^ | **6.7** | **0.362 ± 0.010** | **0.098 ± 0.001** | **0** | **3.7** |
| T11S^b^ | 1.8 | 1.00 ± 0.06 | 0.581 ± 0.007 | -1.1 | 1.7 |
| T11V^b^ | 4.7 | 0.378 ± 0.009 | 0.065 ± 0.001 | 0.7 | 5.8 |
| G17R^b^ | -8.8 | 0.0774 ± 0.0026 | 0.676 ± 0.008 | -5.0 | 29.8 |
| P13R^b^ | 6.7 | 1.80 ± 0.01 | 0.20 ± 0.01 | 1.3 | 9.0 |
| P13H ^b^ | 4.9 | 2.00 ± 0.01 | 0.11 ± 0.01 | 2.3 | 18.2 |
| P13W^b^ | 5.8 | 7.60 ± 0.01 | 0.069 ± 0.001 | 4.9 | 110.1 |
| P13V^b^ | 6.4 | 0.79 ± 0.02 | 0.13 ± 0.01 | 0.7 | 6.1 |
| T11V/G17R^b^ | -7.2 | 0.0164 ± 0.0090 | 0.494 ± 0.028 | -6.8 | 30.1 |
| T11S/G17R^b^ | -7.4 | 0.124 ± 0.013 | 1.06 ± 0.03 | -5.0 | 8.5 |

^a^Values are means (± SD) obtained from three independent experiments.

^b^Data taken from previous publications ^39,49^.

**Table S4**. Oligonucleotides used for site-directed mutagenesis of APPI variants in the pCTCON plasmid

| APPI variant | Template | Primer sequences |
| --- | --- | --- |
| APPI_P13E_ | APPI-3M in pCTCON | Forward primer: 5’ CTGAAACTGGTGAATGTAGAGC 3’  Reverse primer: 5’ CTTGTTCAGAACAAACTTCGC 3’ |
| APPI_A16E_ | APPI-3M in pCTCON | Forward primer:  5’ CATGTAGAGAAGGTTTTTCTAGATG 3’  Reverse primer:  5’ GACCAGTTTCAGCTTGTTC 3’ |
| APPI_G17K_ | APPI-3M in pCTCON | Forward primer:  5’ CATGTAGAGCTAAATTTTCTAGATGG 3’  Reverse primer:  5’ GACCAGTTTCAGCTTGTTC 3’ |
| APPI_R15K_ | APPI-3M in pCTCON | Forward primer: 5’ CATGTAAAGCTGGTTTTTCTAG 3’  Reverse primer: 5’ GACCAGTTTCAGCTTGTTC 3’ |
| APPI_G12D_ | APPI-3M in pCTCON | Forward primer: 5’ CTGAAACTGATCCATGTAGAG 3’  Reverse primer: 5’ CTTGTTCAGAACAAACTTCGC 3’ |
| APPI_T11M_ | APPI-3M in pCTCON | Forward primer: 5’ CTGAAATGGGTCCATGTAGAG 3’  Reverse primer: 5’ CTTGTTCAGAACAAACTTCGC 3’ |
| APPI_T11M,P13I_ | APPI_T11M_ in pCTCON | Forward primer: 5’ CTGAAATGGGTGAATGTAGAGC 3’  Reverse primer: 5’ CTTGTTCAGAACAAACTTCGC 3’ |
| APPI_G12I,G17K_ | APPI_G12I_ in pCTCON | Forward primer:  5’ CATGTAGAGCTAAATTTTCTAGATGG 3’  Reverse primer:  5’ GAATAGTTTCAGCTTGTTCAGAAC 3’ |
| APPI_R15K,A16E_ | APPI_R15K_ in pCTCON | Forward primer:  5’ CATGTAAAGAAGGTTTTTCTAGATG 3’  Reverse primer:  5’ GACCAGTTTCAGCTTGTTC 3’ |

**Table S5**. Oligonucleotides used in site-directed mutagenesis for double mutation APPI variants

| APPI variant | Template | Primer sequences |
| --- | --- | --- |
| APPI_T11R_ | APPI-3M in pPIC9k | Forward primer: 5’ CAAGCTGAAAGAGGTCCATGTAG 3’  Reverse primer: 5’ TTCAGAACAAACTTCGAATTCTTTG 3’ |
| APPI_P13L_ | APPI-3M in pPIC9k | Forward primer: 5’ GAAACTGGTCtATGTAGAGCTG 3’  Reverse primer: 5’ GAAGTTTGTTCTGAACAAGCT 3’ |
| APPI_T11R,G17V_ | APPI_T11R_ in pPIC9k | Forward primer: 5’ ATGTAGAGCTGTTTTTTCTAGATG3’  Reverse primer: 5’ GACCTCTTTCAGCTTGTTCAG 3’ |
| APPI_T11R,F18L_ | APPI_T11R_ in pPIC9k | Forward primer: 5’ GTAGAGCTGGTTTGTCTAGATGG 3’  Reverse primer: 5’ ATGGACCTCTTTCAGCTTG 3’ |
| APPI_P13L,F18L_ | APPI_P13L_ in pPIC9k | Forward primer: 5’ GTAGAGCTGGTTTGTCTAGATGG 3’  Reverse primer: 5’ ATAGACCAGTTTCAGCTTGTT 3’ |
| APPI_P13L,G17S_ | APPI_P13L_ in pPIC9k | Forward primer: 5’ TATGTAGAGCTAGTTTTTCTAGATG 3’  Reverse primer: 5’ ACCAGTTTCAGCTTGTTC 3’ |

**Table S6**. Oligonucleotides used to add NheI and BamHI restriction sites to the edges of the APPI gene sequence

| Source plasmid | APPI variant | Primer sequences for all reactions |
| --- | --- | --- |
| pUC-57 | APPI_T11M,P13N_ APPI_T11M,P13D_ APPI_G17Y,F18K_ APPI_G17H,F18R_ APPI_G17A,F18R_ APPI_F18K,G12I_ APPI_R15A_ APPI_P13I_  APPI_F18R_ APPI_T11M_ APPI_A16G_ APPI_T11M,P13E_ APPI_R15A,A16G_ APPI_T11S_ APPI_G17R_ APPI_T11S,G17R_ APPI_T11V,G17R_ | Forward primer:  5’ ATCTAGCTAGCGAAGTTTGTTCTGAACAAG 3’  Reverse primer:  5’ ATCGCGGATCCAATAGCAGAACCACAAAC 3’ |
| pPIC9K | APPI_P13L,G17S_ APPI_P13L-F18L_ APPI_T11R F18L_ APPI_T11R-G17V_ APPI_T11I-G17S_ APPI_T11S-A16S_ |  |

**Table S7**. Oligonucleotides used to add the APPI gene sequence edges with EcoRI and AvrII restriction sites.

| APPI variant | Template | Primer sequences |
| --- | --- | --- |
| APPI_T11K,P13I_ | APPI_T11K,P13I_ in pCTCON | Forward primer: 5’ CCGGAATTCGAAGTTTGTTCTGAACAAGC 3’  Reverse primer : 5’ TGTCCTAGGAATAGCAGAACCACAAACAG 3’ |
| APPI_G17Y,F18K_ | APPI_G17Y,F18K_ in pCTCON |  |

**References**

1. Cohen I, Kayode O, Hockla A, Sankaran B, Radisky DC, Radisky ES, Papo N (2016) Combinatorial protein engineering of proteolytically resistant mesotrypsin inhibitors as candidates for cancer therapy. Biochemical Journal 473:1329–1341.

2. Lõoke M, Kristjuhan K, Kristjuhan A (2011) Extraction of genomic DNA from yeasts for PCR-based applications. BioTechniques 50:325–328.

3. Chao G, Lau WL, Hackel BJ, Sazinsky SL, Lippow SM, Wittrup KD (2006) Isolating and engineering human antibodies using yeast surface display. Nat Protoc 1:755–768.

4. Salameh MA, Soares AS, Hockla A, Radisky DC, Radisky ES (2011) The P2′ residue is a key determinant of mesotrypsin specificity: engineering a high-affinity inhibitor with anticancer activity. Biochemical Journal 440:95–105.

5. Salameh MA, Soares AS, Hockla A, Radisky ES (2008) Structural Basis for Accelerated Cleavage of Bovine Pancreatic Trypsin Inhibitor (BPTI) by Human Mesotrypsin *. Journal of Biological Chemistry 283:4115–4123.

6. Sananes A, Cohen I, Allon I, Ben-David O, Abu Shareb R, Yegodayev KM, Stepensky D, Elkabets M, Papo N (2023) Serine protease inhibitors decrease metastasis in prostate, breast, and ovarian cancers. Molecular Oncology 17:2337–2355.

7. Naftaly S, Cohen I, Shahar A, Hockla A, Radisky ES, Papo N (2018) Mapping protein selectivity landscapes using multi-target selective screening and next-generation sequencing of combinatorial libraries. Nat Commun 9:3935.

8. Laskowski M, Sealock RW 11 Protein Proteinase Inhibitors—Molecular Aspects. In: Boyer PD, editor. The Enzymes. Vol. 3. Hydrolysis: Peptide Bonds. Academic Press; 1971. pp. 375–473. Available from: https://www.sciencedirect.com/science/article/pii/S1874604708604023

9. Cohen I, Naftaly S, Ben-Zeev E, Hockla A, Radisky ES, Papo N (2018) Pre-equilibrium competitive library screening for tuning inhibitor association rate and specificity toward serine proteases. Biochemical Journal 475:1335–1352.
